# β-Catenin Drives Butyrophilin-like Molecule Loss and γδ T-cell Exclusion in Colon Cancer

**DOI:** 10.1158/2326-6066.CIR-22-0644

**Published:** 2023-07-10

**Authors:** Toshiyasu Suzuki, Anna Kilbey, Nuria Casa-Rodríguez, Amy Lawlor, Anastasia Georgakopoulou, Hannah Hayman, Kyi Lai Yin Swe, Anna Nordin, Claudio Cantù, Pierre Vantourout, Rachel A. Ridgway, Ryan M. Byrne, Lei Chen, Michael P. Verzi, David M. Gay, Ester Gil Vázquez, Hayley L. Belnoue-Davis, Kathryn Gilroy, Anne Helene Køstner, Christian Kersten, Chanitra Thuwajit, Ditte K. Andersen, Robert Wiesheu, Anett Jandke, Karen Blyth, Antonia K. Roseweir, Simon J. Leedham, Philip D. Dunne, Joanne Edwards, Adrian Hayday, Owen J. Sansom, Seth B. Coffelt

**Affiliations:** 1 Cancer Research UK Beatson Institute, Glasgow, United Kingdom.; 2 School of Cancer Sciences, University of Glasgow, Glasgow, United Kingdom.; 3 Wallenberg Centre for Molecular Medicine, Linköping University, Linköping, Sweden.; 4 Department of Biomedical and Clinical Sciences, Linköping University, Linköping, Sweden.; 5 Peter Gorer Department of Immunobiology, School of Immunology & Microbial Sciences, King's College London, London, United Kingdom.; 6 The Francis Crick Institute, London, United Kingdom.; 7 School of Medicine, Dentistry and Biomedical Sciences, Queen's University, Belfast, United Kingdom.; 8 Department of Genetics, Human Genetics Institute of New Jersey, Rutgers Cancer Institute of New Jersey, Rutgers University, New Brunswick, New Jersey.; 9 Nuffield Department of Medicine, Oxford University, Oxford, United Kingdom.; 10 Department of Oncology, Southern Hospital Trust, Kristiansand, Norway.; 11 Department of Research, Southern Hospital Trust, Kristiansand, Norway.; 12 Department of Oncology, Akershus University Hospital, Lørenskog, Norway.; 13 Department of Immunology, Faculty of Medicine Siriraj Hospital, Mahidol University, Nakhon Pathom, Thailand.; 14 BioClavis Ltd., Clydebank, United Kingdom.; 15 School of Medicine, Dentistry & Nursing, University of Glasgow, Glasgow, United Kingdom.

## Abstract

Intraepithelial lymphocytes (IEL) expressing γδ T-cell receptors (γδTCR) play key roles in elimination of colon cancer. However, the precise mechanisms by which progressing cancer cells evade immunosurveillance by these innate T cells are unknown. Here, we investigated how loss of the *Apc* tumor suppressor in gut tissue could enable nascent cancer cells to escape immunosurveillance by cytotoxic γδIELs. In contrast with healthy intestinal or colonic tissue, we found that γδIELs were largely absent from the microenvironment of both mouse and human tumors, and that butyrophilin-like (BTNL) molecules, which can critically regulate γδIEL through direct γδTCR interactions, were also downregulated in tumors. We then demonstrated that β-catenin activation through loss of *Apc* rapidly suppressed expression of the mRNA encoding the HNF4A and HNF4G transcription factors, preventing their binding to promoter regions of *Btnl* genes. Reexpression of BTNL1 and BTNL6 in cancer cells increased γδIEL survival and activation in coculture assays but failed to augment their cancer-killing ability *in vitro* or their recruitment to orthotopic tumors. However, inhibition of β-catenin signaling via genetic deletion of *Bcl9/Bcl9L* in either *Apc*-deficient or mutant β-catenin mouse models restored *Hnf4a, Hnf4g*, and *Btnl* gene expression and γδ T-cell infiltration into tumors. These observations highlight an immune-evasion mechanism specific to WNT-driven colon cancer cells that disrupts γδIEL immunosurveillance and furthers cancer progression.

## Teaser

γδ T cells harbor inherent anticancer activity, but how tumors subvert immunosurveillance by these cells is unclear. The authors identify a mechanism of WNT-driven immune escape from antitumor tissue-resident γδ T cells that is specific to colon cancer.

## Introduction

Intraepithelial lymphocytes (IEL) expressing the γδ T-cell receptor (TCR) account for nearly 50% of all T cells in the mouse gut and 10% to 30% of all T cells in the human intestinal tract. These cells actively migrate in the space between the enterocyte layer and the basement membrane, surveying for abnormalities, including cancer (1, 2). Although diverse, the TCRs of most mouse γδIELs include a Vγ7 chain that facilitates critical interactions with butyrophilin-like (BTNL) molecules, specifically, heterodimers consisting of BTNL1 with BTNL4 or BTNL6 (3–5). Vγ7^+^ cells ordinarily reside only in gut tissue, owing at least in part to the largely restricted expression of BTNL1, BTNL4, and BTNL6 to intestinal epithelial cells (IEC; refs. 3–6). The BTNL1/6 or BTNL1/4 interaction drives Vγ7^+^ IEL expansion and maturation during postnatal development and is thereafter required for maintaining the signature phenotype of Vγ7^+^ IEL (5, 7). The BTNL1/6–γδ T-cell axis in mice is also conserved in humans: human BTNL3 and BTNL8 dimers bind to and regulate Vγ4^+^ IELs (5, 6, 8). The localization of γδ IELs and of BTNL expressions aligns with a decreasing WNT signaling gradient that runs from crypt to villus. As such, Vγ7^+^ IELs are rarely found in the crypt regions where WNT signaling is high.

Most colorectal carcinomas exhibit mutations in members of the WNT pathway that drive tumor initiation and progression to malignancy. These mutations almost exclusively manifest in the form of truncating mutations in the *APC* tumor-suppressor gene, preventing the degradation of β-catenin, which leads to uncontrolled proliferation (9). Like intestinal stem cells residing in crypt regions, colon cancer cells require WNT signaling to maintain their stemness and de-differentiated phenotype (10, 11). However, the relationship between dysregulated WNT signaling in cancer and local, tissue-resident IELs remains wholly unexplored.

Here, we investigated Vγ7^+^ IEL function and the expression of BTNL molecules during tumor initiation and growth. We found that β-catenin signaling in IECs decreased expression of *Btnl* genes and the transcription factors that regulate them, HNF4A and HNF4G. This molecular rewiring also promoted γδ T-cell exclusion from tumors. Conversely, inhibition of β-catenin signaling restored HNF4 transcription factor expression, *Btnl1* gene expression and intratumoral γδ T-cell infiltration. However, reexpression of BTNL1 and BTNL6 in cancer cells was not sufficient to increase Vγ7^+^ IEL infiltration into tumors. Collectively, our data suggest that aberrant WNT signaling in tumors elicits disarray in the tissue-resident γδ T-cell compartment, disrupting natural tissue immunosurveillance as cancer cells dedifferentiate and acquire stem cell–like characteristics.

## Materials and Methods

### Mice

Animal experiments were approved by the UK Home Office (70/8645 and PP6345023 to Karen Blyth, 70/8646, PP3908577 to Owen Sansom, P0B63BC4D to Simon Leedham) and Institutional Animal Care and Use Committees at the Universities of Glasgow and Oxford. Animal experiments were carried out in line with the Animals (Scientific Procedures) Act 1986 and the EU Directive 2010. All mice were maintained on the C57BL/6J background at the Cancer Research UK Beatson Institute, except *Vil1-Grem1* mice and *Lgr5-Cre^ERT2^;Rspo3^INV^* mice, which were maintained at the Functional Genetics Facility, Wellcome Center for Human Genetics, University of Oxford. Mice were bred and housed in individually ventilated cages under specific pathogen-free conditions on a 12/12-hour light/dark cycle and fed and watered *ad libitum*. Both male and female mice at least 6 weeks old and ≥20 kg were used for experiments.

Mice with the following genetic modifications were as follows: *Vil1-Cre^ERT2^* (RRID:IMSR_JAX:020282), *Apc^580^* (RRID:MGI:1857966), *Kras^G12D^* (RRID:MGI:2429948), *Bcl9^F/F^* (RRID:MGI:4398979), *Bcl9l^F/F^* (RRID:MGI:4398981), *Ctnnb1^ex3/+^* (RRID: MGI:1858008), *Lgr5-Cre^ERT2^* (RRID:IMSR_JAX:008875). *Rspo3^INV^* mice were obtained from John Hilkens (Netherlands Cancer Institute; ref. 12). *Vil1-Grem1* mice were generated by Simon Leedham (University of Oxford; ref. 13). *Btnl1^—/—^* mice were generated by Adrian Hayday (Francis Crick Institute; ref. 5). The generation of *Vil1-Cre^ERT2^;Apc^F/+^* (VA) mice, *Vil1-Cre^ERT2^;Apc^F/+^;Kras^G12D/+^* (VAK) mice, *Vil1-Cre^ERT2^;Apc^F/F^* (VA^F/F^) mice, *Vil1-Cre^ERT2^;Apc^F/F^;Kras^G12D/+^* (VA^F/F^K) mice, VA;*Bcl9^F/F^;Bcl9l^F/F^* mice, V;*Ctnnb1^ex3/+^* mice, and V;*Ctnnb1^ex3/+^;Bcl9^F/F^;Bcl9l^F/F^* mice has been described previously (14–17). Generation of *Lgr5-Cre^ERT2^;Rspo3^INV^* mice has been previously described (12). Cre-negative mice were used as controls. Recombination in these tumor models was induced by a single intraperitoneal injection of 80 mg/kg tamoxifen (Sigma-Aldrich T5648–1G), except for VA^F/F^ mice, which 2 injections of 80 mg/kg tamoxifen on 2 consecutive days. Mice were aged until they showed clinical signs (i.e., anemia, hunching and/or weight loss). Tumors were scored macroscopically after fixation of opened intestinal tissue. Tumor burden was calculated by summing the area of all tumors. VA^F/F^ mice were sacrificed 4 days after the first tamoxifen injection, and VA^F/F^K mice were sacrificed 3 days after tamoxifen injection. Recombination in *Lgr5-Cre^ERT2^;Rspo3^INV^* mice was induced by intraperitoneal injection of 1-mg tamoxifen for 5 consecutive days. The porcupine inhibitor LGK-974 was administered by daily oral gavage at 1 mg/kg in 0.5% hydroxypropyl methylcellulose.

For CT26 experiments, female BALB/c mice aged 6 weeks were purchased from Charles River Laboratories. BALB/c mice were used as source for Vγ7^+^ cells for coculture experiments (below). At 8 weeks old, mice were injected with a total of 5×10^5^ CT26 (carrying empty pLIX401 vector) or CT26-B1/6 cells (carrying *Btnl1*-pLIX401-puromycin and *Btnl6*-pLIX401-blasticidin vectors as described below) in PBS subcutaneously into the left flank. Doxycycline (1 mg/mL DOX in 2% sucrose; Sigma-Aldrich D9891–25G) was added to drinking water on day of CT26 injection and provided until endpoint. Mice were monitored three times per week, and tumors were measured by calipers. Humane endpoint was defined as tumors reaching 15 mm in any direction. For orthotopic transplantation, a total of 1×10^6^ CT26 (carrying empty pLIX401 vector) or CT26-B1/6 cells in 70-μL PBS were injected into the submucosal layer of the colon of 10-week-old BALB/c mice, using a Karl Storz TELE PACK VET X LED endoscopic video unit as previously described (18). Doxycycline (1 mg/mL DOX in 2% sucrose) was added to drinking water 7 days after CT26 injection and provided until endpoint. Mice were monitored for weight loss and paling 3 days per week. Humane endpoint was defined as weight loss ≥20%.

### Cell lines

CT26 cells were maintained in RPMI-1640 medium (Thermo Fisher Scientific 31870074) with 10% heat-inactivated FCS (Thermo Fisher Scientific 15808947), 100 U/mL penicillin/streptomycin (Thermo Fisher Scientific 15140148), 2 mmol/L l-glutamine (Thermo Fisher Scientific 25030081), 50 μmol/L β-mercaptoethanol (Thermo Fisher Scientific 21985023), 1 mmol/L sodium pyruvate (Thermo Fisher Scientific 11360088). MODE-K and HEK-293T cells were maintained in DMEM, 4.5 g/L D-glucose with GlutaMAX (Thermo Fisher Scientific 31331093), supplemented with 10% heat-inactivated FCS and 1% penicillin–streptomycin. Cells were kept at 37°C and 5% CO_2_. CT26 cells were obtained from Stephen Tait (University of Glasgow, UK) in 2021. HEK-293FT cells (RRID:CVCL_HA71) were obtained from James Neil (University of Glasgow, UK) in 2017. MODE-K cells were obtained from Dominique Kaiserlain (University of Lyon, France) in 2014. No authentication was performed. *Mycoplasma* testing was performed monthly. After thawing, cells were not used past 15 passages.

### Overexpression of transcription factors in MODE-K cells

The murine transcription factors *Cdx1*, *Cdx2*, *Creb3l3*, *Gata5*, *Hnf4a*, and *Isx* were cloned into pCSIGPW [lentiviral vector bearing an IRES-GFP reporter and resistance to puromycin, described in ref. (5)] by conventional RT-PCR, using cDNA derived from C57BL/6 IECs as template and the following primers (lowercase symbols correspond to spacers and restriction sites): *Cdx1* forward 5′-atatgaattcCCCTGCGGTCACCATG-3′, reverse 5′-atatctcgagCAGGCTGCAAGGGGCTAG-3′; *Cdx2* forward 5′-atatgaattcGTCCCTCGCCACCATG-3′, reverse 5′-atatctcgagCCACGGGAGGGGTCAC; *Creb3l3* forward 5′-atatctcgagCAGGCACGGGACTCATG-3′, reverse 5′-atatgcggccgcAGGGCTGTCTGAGTCTGTCAC-3′; *Gata5* forward 5′-atatctcgagCGCGGGGAAAAAAAATG-3′, reverse 5′-atatgcggccgcGTGACAGTTTCCTGAGCACCTAG-3′; *Hnf4a* forward 5′-atatgaattcCGTGGGTAGGGGAGAATG-3′; reverse 5′-atatgcggccgcCCCCAGCAGCTTGCTAG-3′; *Isx* forward 5′-atatgaattcAGCAGGGCTAGGCCATG-3′; reverse 5′-atatgcggccgcGCTGCCTGCCATCAC-3′. All plasmids used for lentiviral transduction were purified using a NucleoBond Xtra Midi EF kit (Macherey-Nagel 740420.50). Lentiviral particles were produced in HEK-293T cells by cotransfection of pCSIGPW either empty vector (EV) or encoding the indicated murine transcription factors, HIV-1 gag-pol pCR/V1 (19) and VSV-G env pHIT/G (20). Medium was replaced 16 hours after transfection and was collected at 48 hours, filtered through 0.45-μm cellulose acetate mesh, and then added to subconfluent MODE-K cells grown in 24-well plates. After 24 hours, MODE-K cells were transferred to 12-well plates in DMEM, 4.5 g/L D-glucose with GlutaMAX, 10% heat-inactivated FCS, 1% penicillin–streptomycin, supplemented with 1 μg/mL puromycin (Merck P8833–25MG), and selected for 2 weeks. Where necessary, cells were sorted on the basis of GFP expression to homogenize expression across lines. Expression of *Btnl1*, *Btnl4, Btnl6*, and *Gapdh* (positive control for the presence of cDNA) was evaluated in MODE-K lines transduced with the indicated transcription factors or the EV control (above) by conventional RT-PCR, using the following primers: *Btnl1* forward 5′-GACTTGACCTTCCACTCTGATG-3′, reverse 5′-ATTCCCTGTGCACATCACTTAG-3′; *Btnl4* forward 5′-ATGGAAAATCACCGCAAGCCC-3′, reverse 5′-CTTCTACATTCCCACAAGGAGC-3′; *Btnl6* forward 5′-ATGGAAAATCACCGCAAGCCA-3′, reverse 5′-CTAACTTCTTCCCATTCTTCCC-3′; *Gapdh* forward 5′-GTAGACAAAATGGTGAAGGTCG-3′, reverse 5′-GACTCCACGACATACTCAGCAC-3′. cDNA derived from C57BL/6 IECs was used as a positive control of *Btnl* expression.

### Generation of *Btnl1/Btnl6*-expressing CT26 cancer cells

Mouse *Btnl1* and *Btnl6* cDNA was purchased from Invitrogen in pMA-T and pMK-RQ plasmids, respectively. DNA fragments of *Btnl1* and *Btnl6* with NheI and SbfI were inserted into the doxycycline-inducible plasmid pLIX401 with puromycin resistance (Addgene 41393-DNA.cg). To generate a blasticidin-resistant *Btnl6*-pLIX401 plasmid, a partial PGK promoter followed by a blasticidin-resistant gene in pMK-RQ, and the puromycin-resistant gene was replaced with the blasticidin-resistant gene at AscI and XbaI sites. Viral supernatants were prepared as before (21), following transient transfection of HEK-293FT cells with empty pLIX401, *Btnl1*-pLIX401-puromycin, or *Btnl6*-pLIX401-blasticidin together with pSPAX2 packaging vector (RRID:Addgene_12260) and pVSVG envelope vector (RRID:Addgene_85140) using Lipofectamine 2000 (Thermo Fisher Scientific, 11668019). CT26 cells were transduced with empty pLIX401, *Btnl1*-pLIX401-puromycin, or *Btnl6*-pLIX401-blasticidin viral supernatants, and then expanded in 6 μg/mL puromycin or 8 μg/mL blasticidin (Thermo Fisher Scientific A1113903), as appropriate. Stable CT26 cells containing *Btnl1*-pLIX401-puromycin vector were then transduced with *Btnl6*-pLIX401-blasticidin viral supernatants, and CT26 cells containing *Btnl6*-pLIX401-blasticidin vector were then transduced with *Btnl1*-pLIX401-puromycin. These cells were expanded in both 6 μg/mL puromycin and 8 μg/mL blasticidin to generate CT26 cells, expressing both *Btnl1* and *Btnl6*. Confirmation of *Btnl1* and *Btnl6* mRNA expression was performed by qRT-PCR as described below after varying doses of doxycycline for 24 hours.

### IHC and ISH

Tissues from Cre-negative, VA, VA;*Btnl1*^—/—^, VAK, VA^F/F^, VA^F/F^K, VA;*Bcl9^F/F^;Bcl9l^F/F^*, V;*Ctnnb1^ex3/+^*, V;*Ctnnb1^ex3/+^;Bcl9^F/F^;Bcl9l^F/F^*, *Vil1-Grem1*, and *Lgr5-Cre^ERT2^;Rspo3^INV^* mice as well as CT26 tumors were fixed overnight in 10% neutral-buffered formalin, then embedded in paraffin. Staining was performed on 4-μm sections that had been heated at 60°C for 2 hours. Primary antibodies used for IHC were as follows: CDX1 (1:250; Invitrogen #PA5–23056), CDX2 (1:200; Abcam #ab76541), HNF4A (1:10,000; Perseus Proteomics #pph1414–00), HNF4G (1:1,000; Novus Biologicals #NBP1–82531), and SOX9 (1:500; Millipore #AB5535). HNF4A and SOX9 were detected by an Agilent AutostainerLink48 using high pH citrate buffer (Target Retrieval Solution, Aglient #K8004/K8005) and peroxidase blocking. CDX1, CDX2, and HNF4G were detected on a Leica Bond Rx autostainer, using ER2 antigen retrieval solution (Leica #AR9640). For RNAscope, the following probes were used from Advanced Cell Diagnostics: *Btnl1* (436648) and *Trdc* (449358). Staining was performed on a Leica Bond Rx autostainer according to Advanced Cell Diagnostics instructions. Images were acquired with an Olympus BX51 or Zeiss Axio Imager.A2 microscope. For each antibody or RNAscope probe, staining was performed on tissue sections from at least three mice of each genotype, and representative images are shown for each staining. The average number of γδ T cells was determined by HALO image analysis software (Indica Labs, CytoNuclear v1.5 algorithm) in 10^6^ μm^2^ tissue from 1 to 5 villi or tumors within each mouse.

### Gene expression analysis of mouse tissue

RNA sequencing (RNA-seq) data from wild-type (WT), VA^F/F^, and VA^F/F^K mouse intestinal tissue were generated for previous studies (ArrayExpress E-MTAB-7546; refs. 14, 15, 22). In this study, we performed analysis of these data as previously described where raw counts per gene were determined using FeatureCounts (RRID:SCR_012919) version 1.6.4 (22). Differential expression analysis was performed using the R package DESeq2 (RRID:SCR_000154) version 1.22.2, and principal component analysis was performed using R base functions. RNA-seq data from WT, *Hnf4a^Δ/Δ^*, *Hnf4g^Δ/Δ^* and *Hnf4a^Δ/Δ^;Hnf4g^Δ/Δ^* mice were analyzed as previously described (23); these data were obtained from GSE112946.

### Flow cytometry

Tumors and 1 cm^2^ of jejunum from Cre-negative, VA, and VAK mice were cut into small pieces using the McIlwain Tissue Chopper and digested on the gentleMACS Octo Dissociator with Heaters (program, 37C_m_TDK_1) using the mouse Tumor Dissociation Kit (Miltenyi Biotec 130–096–730) according to the manufacturer's instructions, and prepared cells were resuspended in 0.5% BSA in PBS. Cells were stained in the brilliant stain buffer (BD Biosciences 566349) containing antibodies for 30 minutes at 4°C in the dark. The following antibodies were used: CD19-APC-eFluor780 (clone 1D3; 1:400; eBioscience 47–0193–82), CD3ε-BV650 (clone 17A2; 1:100; BioLegend 100229), CD8α-BUV805 (clone 53–6.7; 1:50; BD Biosciences 564920), EpCAM-APC-eFluor780 (clone G8.8; 1:100; eBioscience 47–5791–82), and TCRδ-FITC (clone GL3; 1:200; eBioscience 11–5711–85). Dead cells were identified with Zombie NIR Fixable Viability dye (BioLegend 423106). Cells were acquired using a 5-laser BD LSRFortessa flow cytometer with DIVA software (BD Biosciences). Data were analyzed using FlowJo Software (RRID:SCR_008520) version 9.9.6.

### Human patient cohorts and IHC

The Scotland cohort of paraffin-embedded colon tumors was assembled from 1,030 patients who had undergone a resection for Stage I to IV colon cancer between 1997 and 2007 at the Glasgow Royal Infirmary, Western Infirmary or Stobhill Hospital in Glasgow, UK. The West of Scotland Research Ethics Committee granted study approval (16/WS/0207). Patient information and tissue blocks were held within the Glasgow and Clyde Safe Haven (12/WS/0142). Tumors were staged using the 5th edition of AJCC/UICC-TNM staging system. A subcohort of 144 samples was selected for IHC, and tissue was available from 142 patients where both tumor and normal adjacent tissue was visible. The Norway cohort was assembled from 299 patients who had undergone a resection for Stage II to III colon cancer between 2000 and 2020 at the Southern Hospital Trust in Norway. The Sørlandet Hospital Ethics Committee granted study approval. Tumors were staged using the 5th edition of AJCC/UICC-TNM staging system from 2000 to 2009, the 7th edition from 2010 to 2017, and the 8th edition thereafter. A subcohort of 84 samples was selected for IHC, and tissue was available from 71 patients where both tumor and normal adjacent tissue was visible. The Thailand cohort was assembled from 411 patients who had undergone a resection for Stage I to IV colon cancer between 2009 and 2016 at hospitals in Thailand. These samples were approved by the Siriraj Institution Review Board (COA no.Si544/2015). Tumors were staged using the 6th or 7th editions of AJCC/UICC-TNM staging system. A subcohort of 136 samples was selected for IHC, and tissue was available from 122 patients where both tumor and normal adjacent tissue was visible. Across all cohorts, tissues were assembled retrospectively so no patient consent was obtained, and samples were excluded if patients had received neoadjuvant chemotherapy or died within 30 days of surgery. These studies were conducted in accordance with the ethical principles of the World Medical Association Declaration of Helsinki. Clinicopathological characteristics are listed in Supplementary Table S1.

IHC was performed on full tissue sections with citrate buffer (pH 6.0) antigen retrieval with standard protocols, using an anti-TCRδ antibody (1:300; clone H-41, Santa Cruz Biotechnology #sc-100289, lot K1318 or K2618) previously validated (24). Scoring of γδ T cells was conducted using VisioPharm software. The first level of tissue compartments (primary tumor, adjacent normal tissue) was manually annotated. A tissue classifier was built using RGB and hematoxylin features with the application of a K-means clustering algorithm and was trained using sections from all cohorts. A pan-lymphocyte detector was built using a five-pixel mean filter applied to the chromogenic DAB feature and a dual hematoxylin feature consisting of a polynomial smoothing filter and a polynomial Laplace filter at a field size of 15 pixels at an order of two. The output metric is defined as the percentage of total cells within an analyzed region that are positively identified as the target cell type.

### Gene expression analysis in human tumors

TempO-Seq whole-transcriptome profiling was performed on 82 patients from the Scotland cohort, according to the manufacturer's instructions using whole formalin-fixed, paraffin-embedded (FFPE) tissue sections. 77 out of the 82 had matched γδ T-cell IHC data. FFPE tissue was deparaffinized before tissue digestion. Crude tissue lysates were used as input for whole-transcriptome analysis using the Human Whole Transcriptome v2.0 panel (Biospyder Technologies). Detector oligos, consisting of a sequence complementary to an mRNA target plus a universal primer landing site, were annealed in immediate juxtaposition to each other on the targeted RNA template and ligated (25). Amplification of ligated oligos was performed using a unique primer set for each sample, introducing a sample-specific barcode and Illumina adaptors. Barcoded samples were pooled into a single library and run on an Illumina HiSeq 2500 High Output v4 flowcell. Sequencing reads were demultiplexed using BCL2FASTQ software (Illumina). FASTQ files were aligned to the Human Whole Transcriptome v2.0 panel, which consist of 22,537 probes, using STAR (26). Up to two mismatches were allowed in the 50-nucleotide sequencing read. Deseq2 was used to normalize raw read counts. Linear regression analysis on paired samples was performed using Prism software (version 9.3.1).

Oncomine (oncomine.org) was used to query gene expression levels of *BTNL3, BTNL8, HNF4A, HNF4G, CDX1*, and *CDX2* in microarray analyses from the TCGA (9) and Skrzypczak (27) cohorts. The TCGA dataset consisted of 19 normal and 101 tumor tissues. The Skrypczak dataset consisted of 24 normal and 45 tumor tissues. Median, minimum, maximum expression levels were extracted from Oncomine analysis and imported into Prism software (version 9.3.1) for visualization. Expression levels are presented as log_2_ median-centered intensity.

The Marisa cohort consists of fresh-frozen primary tumor samples from patients with colon cancer collected and transcriptionally profiled as described previously (28). The normalized, batch corrected microarray data for the Marisa cohort were downloaded from Gene Expression Omnibus (GEO; RRID:SCR_005012) using the accession number GSE39582. This dataset had been processed using the Robust Multi-Array Analysis method and corrected for technical batch effects using ComBat as described previously (28). Probesets were collapsed to the gene level by selecting the probeset with the highest mean expression value across all samples for each gene using the collapseRows function (method = “MaxMean”) from the WGCNA package (29) using R (v3.3.2). Only tumor samples from patients with Stage II or III disease who did not receive adjuvant chemotherapy that had relapse-free survival data (*n* = 258) were used analysis.

### Organoid culture and treatment

Small intestine was harvested from mice of indicated genotypes. Organoids were generated as previously described (15, 22), cultured in Matrigel (Corning, 356231) with ENR medium [Advanced DMEM/F12 containing 2 mmol/L Glutamine, 10 mmol/L HEPES, 1× N2 supplement (Thermo Fisher Scientific 17502048), 1× B27 supplement (Thermo Fisher Scientific 12587010), 50 ng/mL EGF (PeproTech AF-100–150), 100 ng/mL Noggin (PeproTech 250–38), 1,000 ng/mL R-spondin 1 (PeproTech 120–38), 100 U/mL of penicillin, and 100 U/mL of streptomycin]. Organoids were split every 2 to 3 days. Where indicated, organoids from WT (Cre-negative mice), VA^F/F^, VK, and VA^F/F^K mice were treated with 1 μmol/L 4-Hydroxytamoxifen (4-OHT, Sigma T5648) in ENR medium for 48 hours. Organoids from Cre-negative mice were treated with 3 and 10 μmol/L CHIR-99021 (Sigma SML1046) or DMSO as a control (1:300 dilution) in ENR medium for 6 days. Medium containing CHIR-99021 (GSK3β inhibitor) was changed every day. After 6 days, organoids were cultured in ENR medium without CHIR-99021 or DMSO for 2 days. Organoids from Cre-negative mice were treated with 100 μmol/L BI-6015 (Cayman CAY12032) or DMSO as a control (1:100 dilution) in ENR medium for 3 days. Cells were collected for downstream analysis on indicated day after treatment. Biological replicates were generated from individual mouse organoid lines.

Short hairpin (sh)RNA target sequences designed against *Cdx1, Cdx2, Hnf4a, Hnf4g*, and *Sox9* were selected from Merck Mission shRNAs (https://www.sigmaaldrich.com/GB/en/product/sigma/shclnd). 5 sequences per gene were subcloned into the pLKO.1-Puro lentiviral backbone (Addgene #8453), and inserts sequenced before use. Viral supernatants were prepared following transient transfection of 293FT cells with pLKO.1 encoding shRNAs, pSPAX2 packaging vector and pVSVG envelope vector using Lipofectamine 2000 as described previously (21). Two 24-hour supernatants were collected sequentially over a 48-hour period, pooled and filtered through a 0.45-μm syringe filter and then concentrated using the Lenti-X Concentrator solution (Clontech/Takara). Intestinal WT organoids were expanded 3 days before infection in normal growth medium supplemented with 1 μg/mL R-Spondin, 3 μmol/L CHIR-99021, 10 μmol/L Y27632 (ROCK inhibitor, Cambridge Bioscience SM02–1), and 1 μmol/L Jagged-1 (Notch Ligand 1, Cambridge Bioscience 188–204) to enrich stem and progenitor cells (30). VA^F/F^ organoids that received *Sox9* shRNAs were similarly expanded but no supplements were added. Organoids were reseeded into the same medium 24 hours before infection. Freshly concentrated viral supernatants were added directly to harvested, manually disrupted organoids in the presence of 8 μg/mL polybrene (Merck TR-1003-G) and mixtures seeded into 12-well plates coated with a fine film of Matrigel. Organoid fragments were left to attach overnight and then drained before overlaying with a fine film of Matrigel. Organoids were expanded in culture medium as above, supplemented with 1 μg/mL R-Spondin (WT organoids only) and 3 μg/mL puromycin.

### Quantitative RT-PCR

RNA was isolated from fresh intestinal organoids using the Qiagen's RNeasy kit (74106) with on-column DNA digestion. RNA concentration and purity (cutoff = 2.0–2.2 260/280 ratio) was determined using a Thermo Scientific NanoDrop spectrophotometer with NanoDrop 2000 software. cDNA was prepared from 0.5 to 1 μg RNA using a Quantitect Reverse Transcription Kit (Qiagen 205311) and diluted to 2.5 ng/mL in DEPC-treated water. For quantitative RT-PCR, 12.5 ng aliquots of cDNA were amplified in triplicate on an ABI 7500 real-time PCR machine using SyGreen Blue Mix Lo‐ROX PCR master mix (PCR Biosystems PB20.15–51) and primers (below), all at 2.0 μmol/L except for *Btnl1* (1 μmol/L Fwd; 4 μmol/L Rv), with endogenous controls *Hprt* (Mm_Hprt_1_SG; Quantitect) and β-actin (Mm_Actn_1_SG; Quantitect). Relative expression was calculated by the Δ*C*_t_ method after averaging endogenous controls. Data are displayed as fold change (2^−ΔΔCt^). The following primer sequences were used for each gene: *Btnl1* forward 5′-CCGGGAACACGCTACTGTC-3′, reverse 5′-CAAACCAGGGCTACTTTCCAT-3′; *Btnl2* forward 5′-TTTGCTATGGATGACCCTGC-3′, reverse 5′-TCCTGATTGCTGCTGTGTGT-3′; *Btnl4* forward 5′-CATTCTCCTCAGAGACCCACACTA-3′, reverse 5′-GAGAGGCCTGAGGGAAGAA-3′; *Btnl6* forward 5′-CGTGTGGAGGATAATAAGGCAGA-3′, reverse 5′-TCCTTGCGCCAATCTGCATAC-3′. The other primers were purchased from QIAGEN (Quantitect Primer): *Hprt* (QT00166768); *Axin2* (QT00126539); *Lgr5* (QT00123193); *Sox9* (QT00163765); *Cdx1* (QT00265139): *Cdx2* (QT00116739); *Cd44* (QT00173404); *Hnf4a* (QT00144739); *Hnf4 g* (QT00169799).

### Gene promoter analysis

Promoter sequences for mouse *Btnl1*, *Btnl2*, *Btnl4*, and *Btnl6* and human *BTNL3* and *BTNL8* (12 kb upstream of ATG start site) were extracted from the UCSC Genome Browser (31). These sequences were analyzed by The Open Regulatory Annotation database (ORegAnno; ref. 32) for putative transcription factor–binding sites using ApE software.

### Chromatin immunoprecipitation sequencing

Chromatin immunoprecipitation (ChIP)-seq data were generated previously (23) on 3 mice per group using anti-HNF4A (6 μg, Santa Cruz Biotechnology #sc-6556 X, lot B1015) and anti-HNF4G (6 μg, Santa Cruz Biotechnology #sc-6558 X, lot F0310) antibodies. ChIP-seq data were obtained from GSE112946, analyzed as described previously (23), and visualized using IGV (33).

### Cleavage under targets and release using nuclease, ATAC-seq, and genomic regions enrichment of annotations tool analysis

LEF1 and IgG-negative control cleavage under targets and release using nuclease (CUT&RUN) datasets and ATAC-seq data from three biological replicates of HEK-293T cells were analyzed as described previously (34, 35) and visualized in IGV (33). These data are available from ArrayExpress (E-MTAB-12077 and E-MTAB-12076). Conservation data were obtained from the UCSC genome browser 100 vertebrates’ track (31). ConTra (36) was used to determine JASPAR LEF1 core binding sites (MA0768.1). Genomic regions enrichment of annotations tool (GREAT) annotation analysis (37) was used on default settings to assign genes to genomic coordinates of the LEF1 signal enrichment.

### Coculture assays using γδ T and cancer cells

Murine small intestine (SI) from naive Balb/c mice was isolated and lymph nodes and mesentery were removed. SI was washed with PBS and cut into 1×1-cm pieces and transferred into ice-cold PBS. SI pieces were dissociated in HBSS with 1 mmol/L DTT and 5 mmol/L EDTA at 37°C for 30 minutes with rocking at 75 rpm, after which tissue pieces were physically disrupted by pipetting. SI pieces were then transferred into fresh PBS for further physical disruption by pipetting again. SI pieces were removed and both dissociation solutions were combined, passed through 70-μm strainers and centrifuged to pellet cells. Cell suspension was centrifuged on 20/40/80% Percoll density gradient at 700 × *g* for 30 minutes. Lymphocytes were collected from the interface between 40/80% Percoll. Cells were stained for the following antibodies in Brilliant Stain buffer (BD Biosciences) followed by live dead cell staining using Zombie Violet Fixable Viability kit (BioLegend): NKp46-FITC (clone 29A1.4; 1:100; BioLegend 137606); EpCAM-FITC (clone G8.8, 1:50, eBioscience 11–5791–82); CD11b-BV786 (clone M1/70, 1:200, BioLegend 101203); CD3ε-BV650 (clone 17A2, 1:100, BioLegend 100229); TCRβ-BV510 (clone H57–597, 1:100, BioLegend 109234); CD8α-APC-eFluor780 (clone 53–6.7, 1:50, eBioscience 47–0081–82); Vγ4-PE-Cy7 (clone UC3–10A6, 1:100, BioLegend 137708); CD11c-PE-Cy5 (clone N418, 1:100, eBioscience 15–0114–82); Vγ1-PE (clone 2.11, 1:200, BioLegend 141106); and CD19-PE (clone 1D3/CD19, 1:100, BioLegend 152407). Cells were filtered and transferred into 2% FCS, 25 mmol/L HEPES, 2 mmol/L EDTA in PBS. Vγ7^+^ cells were sorted by negative selection into IMDM medium containing 8% FCS, 100U/mL penicillin streptomycin, 50 μmol/L β-mercaptoethanol using a FACSAria cell sorter with DIVA software (BD Biosciences).

Twenty-four hours before coculture, CT26 and CT26-B1/6 cells were plated into a 96-well plate at a concentration of a total of 2×10^3^ cells per well in RPMI medium containing doxycycline (1 μg/mL). Mouse Vγ7^+^ cells (2×10^4^ cells) were added to CT26 cells in medium containing IL2 (10 IU/mL, PreproTech 212–12), IL3 (100 IU/mL, PreproTech 213–13), IL4 (100 IU/mL, PreproTech 214–14), and IL15 (100 ng/mL, PreproTech 210–15) and doxycycline (1 μg/mL). Cells were cultured for 24 hours at 37°C. After 24 hours, medium was collected, and then CT26 cells were detached by trypsin. Detached CT26 cells and collected medium were combined, centrifuged to form a cell pellet and stained in Brilliant Stain buffer (BD Biosciences) with the following antibodies and analyzed using a BD Fortessa flow cytometer with DIVA software: EpCAM-eFluor780 (clone G8.8, 1:50, eBioscience 47–5791–82); CD3ε-FITC (clone 145–2C11, 1:100, eBioscience 11–0031–86); TCRδ-PE (clone GL3, 1:100, BioLegend 118108); Vγ7-Dylight650 (clone F2.67, 1:100, BioLegend 161702); CD44-PerCP-Cy5.5 (clone IM7, 1:50, BioLegend 103032); CD25-BV711 (clone PC61, 1:100, BioLegend 102049); CD45-BV605 (clone 30-F11, 1:50, eBioscience 83–0451–42); and CD122-PE/Dazzle594 (clone TM-β1, 1:100, BioLegend 123217).

### Statistical analysis

An unpaired *t* test or the nonparametric Mann–Whitney test or paired *t* test was used to compare two groups. One-way ANOVA was used to compare groups of three or more followed by the Tukey or Dunnett *post hoc* test. The log-rank (Mantel–Cox) test was used to analyze Kaplan–Meier survival curves. Correlation between genes was determined using the Pearson correlation coefficient. *P* values less than 0.05 were considered statistically significant. Graphs were generated and statistical significance calculated using Prism software (version 9.3.1). The statistical tests used are indicated in figure legends. For all animal and organoid experiments, each data point represents an individual mouse or individual organoid line.

### Data availability

Data analyzed in this study were obtained from GEO at GSE39582 (RNA-seq and ChIP-seq data) and GSE112946 (microarray data), and ArrayExpress at E-MTAB-7546 (RNA-seq data), E-MTAB-12077 (CUT&RUN-LoV-U data), and E-MTAB-12076 (ATAC-seq data). The Tempo-Seq gene expression data generated in this study are publicly available in ArrayExpress at E-MTAB-13077. All other data generated in this study are available within the article and its Supplementary Data Files or from the corresponding author upon reasonable request.

## Results

### Vγ7^+^ cells suppress gut tumor formation

**Figure 1.**
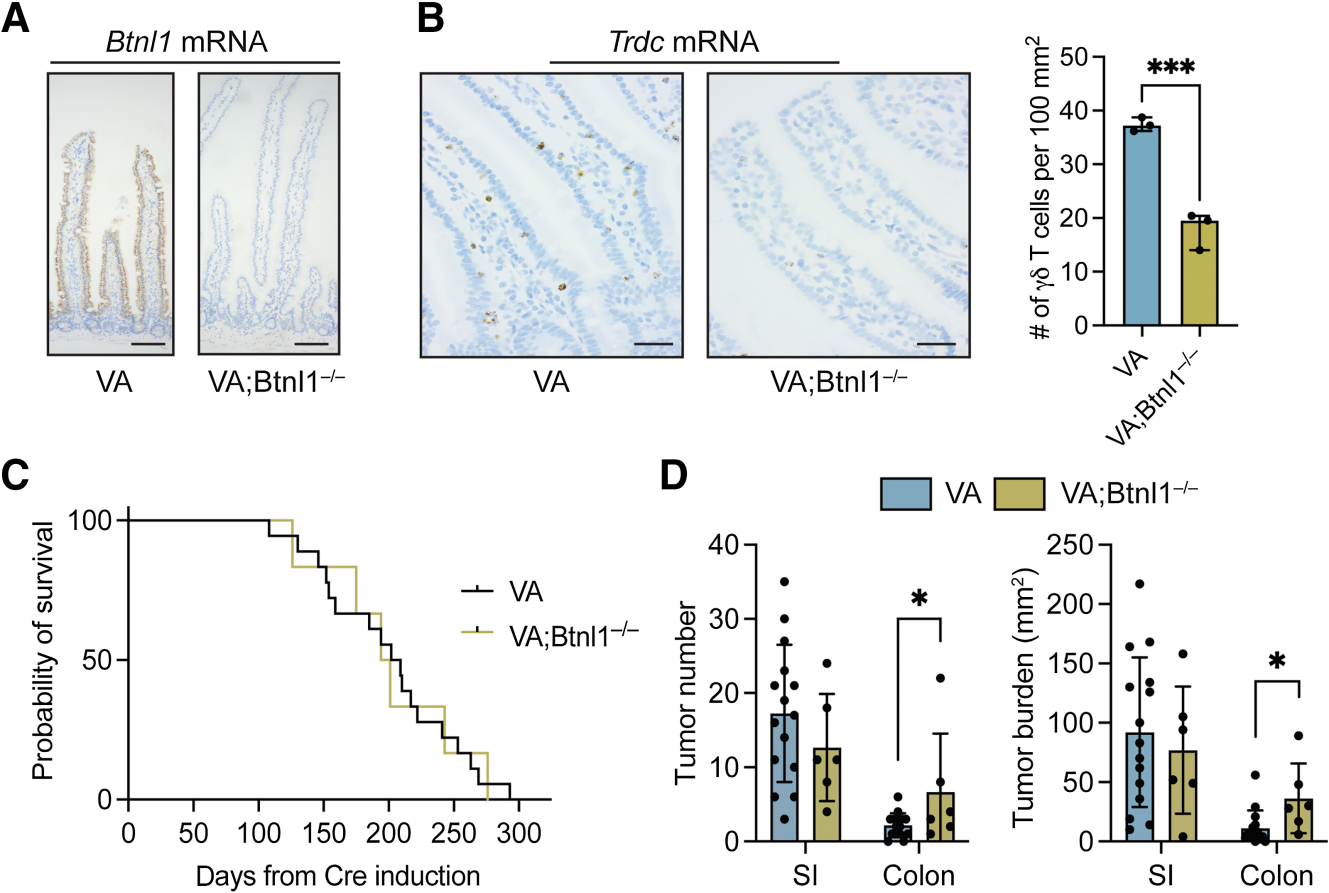
Loss of *Btnl1* increases adenoma formation in *Apc*-deficient mouse models. **A,** Images of SI from VA and VA;*Btnl1^—/—^* mice stained for *Btnl1* mRNA representative of 4/group; scale bar, 100 μm. **B,** Images of SI from VA and VA;*Btnl1^—/—^* mice stained for *Trdc* mRNA; scale bar, 500 μm. γδ T-cell numbers in SI of indicated models. Each dot represents one mouse (*n* = 3). **C,** Kaplan–Meier survival analysis of VA (*n* = 15) and VA;*Btnl1^—/—^* (*n* = 6) mice. **D,** Tumor number and tumor burden (mm^2^) in SI and colon of VA and VA;*Btnl1^—/—^* mice. Each dot represents one mouse (*n* = 15 VA, 6 VA;*Btnl1^—/—^* mice). Data are presented as mean ± SD. *, *P* < 0.05; ***, *P* < 0.001 (unpaired *t* test).

To test the importance of gut-resident Vγ7^+^ cells in tumor initiation and progression, we crossed *Villin-Cre^ERT2^;Apc^F/+^* (VA) mice with *Btnl1^—/—^* mice, which harbor significantly diminished Vγ7^+^ cell compartments in the SI and colon (5). We confirmed that *Btnl1* expression was absent from gut tissue of VA;*Btnl1^—/—^* mice, whereas *Btnl1* expression was maintained in VA mice (Fig. 1A). The number of γδ T cells in normal, tumor-adjacent regions was reduced in VA;*Btnl1^—/—^* mice when compared with VA mice (Fig. 1B). Overall survival of tumor-bearing VA and VA;*Btnl1^—/—^* mice was the same, and there was comparable tumor incidence and burden in the SI of tumor-bearing VA and VA;*Btnl1^—/—^* mice (Fig. 1C and D). Conversely, tumor number and particularly tumor burden were increased in the colon of VA;*Btnl1^—/—^* mice when compared with VA mice (Fig. 1D). The lack of phenotype in the SI may be explained by compensation from cytotoxic TCRαβ^+^ IELs and other γδ T-cell subsets (e.g., Vγ1^+^ cells), which partially offset Vγ7^+^ cell deficiencies in *Btnl1*-deficient mice (5). Because bacterial load is higher in the mouse distal colon than in the SI (38), the propensity for inflammation-driven tumors in this anatomical location may be more sensitive to the lack of Vγ7^+^ cells, which are crucial infection sensors, protectors from pathogens, and suppressors of inflammation (39, 40). However, we cannot rule out any cancer cell–intrinsic effect of *Btnl1* deletion in the VA model. In sum, loss of Vγ7^+^ cells was associated with increased tumorigenesis, in agreement with the established role of Vγ7^+^ cells in cancer immunosurveillance (2).

### Mouse and human tumors exhibit a paucity of γδ T cells

**Figure 2.**
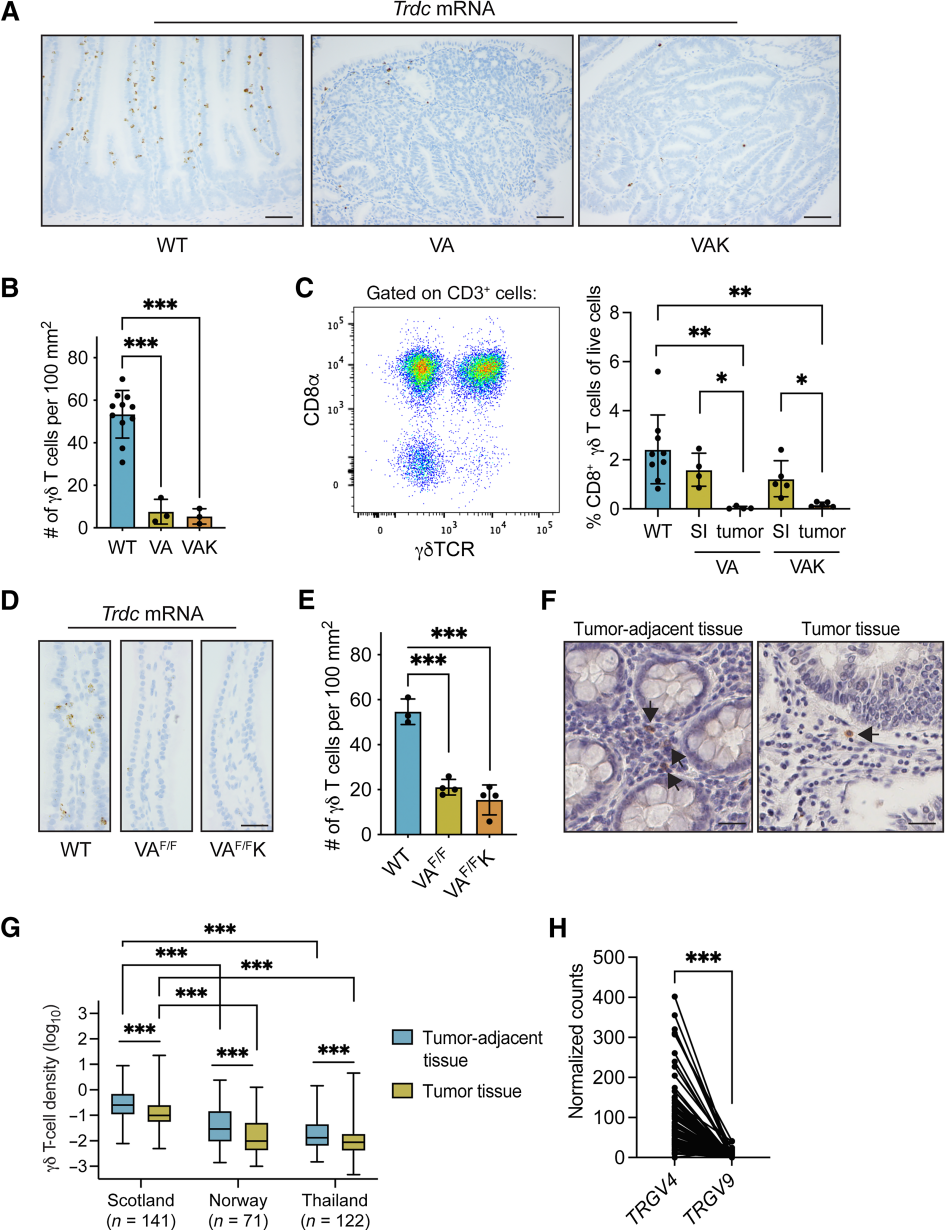
γδ T cells are excluded from mouse and human gut tumors. **A,** Images of SI tissue from 4 WT (Cre-negative), tumor-bearing *Villin-Cre^ERT2^;Apc^F/+^* (VA) and tumor-bearing *Villin-Cre^ERT2^;Apc^F/+^;Kras^G12D^* (VAK) mice stained for *Trdc* mRNA; scale bar, 500 μm. **B,** γδ T-cell numbers in SI tissue of indicated models. Each dot represents one mouse (*n* = 11 WT, 3 VA, 3 VAK). **C,** Representative flow cytometry plot of CD8α and γδTCR expressions on total CD3^+^ cells in the small intestine of WT mice. CD8α^+^ γδ T-cell frequency in SI of indicated models. Each dot represents one mouse (*n* = 9 WT, 4 VA, 5 VAK). **D,** Images of SI from indicated models (*n* = 4/group) stained for *Trdc* mRNA; scale bar, 500 μm. **E,** γδ T-cell numbers in SI of indicated models. Each dot represents one mouse (*n* = 3 WT, 4 VA^F/F^, 4 VA^F/F^K). **F,** Image of γδ T-cell staining in tumor adjacent tissue and tumor tissue from human colon cancer sections (Scotland cohort, *n* = 141) where arrows indicate positively stained cells; scale bar, 500 μm. **G,** Density of γδ T cells in human colon cancer sections in three different patient cohorts: Scotland (*n* = 141), Norway (*n* = 71), and Thailand (*n* = 122). γδ T cells identified by IHC in full sections were quantified in tumor adjacent tissue or tumor tissue using Visiopharm. Data are presented as median ± min/max. **H,** Expressions of *TRGV4* and *TRGV9* mRNA in human colon cancer samples (*n* = 82) from the Scotland cohort determined by TempO-Seq. Data are presented as mean ± SD. *, *P* < 0.05; **, *P* < 0.01; ***, *P* < 0.001 (paired *t* test or one-way ANOVA followed by Dunnett *post hoc* test).

We next asked whether the prevalence of Vγ7^+^ cells in normal gut tissue was maintained in tumors. Contrary to their abundance in gut tissue of WT (Cre-negative) mice, γδ T cells were sparse within adenomas from VA mice, as well as an additional model of colon cancer, *Villin-Cre^ERT2^;Apc^F/+^;Kras^G12D/+^* (VAK) mice (Fig. 2A). The cells’ frequency was estimated at 7- to 10-fold lower than in normal tissue (Fig. 2B). Because Vγ7^+^ IELs express CD8αα dimers, whereas most other intestinal γδ T cells do not (5), we used CD8α as a marker to specifically quantify the Vγ7^+^ cell representation in tumor-bearing VA and VAK mice. CD8α^+^ γδ T cells were apparent in the SI of WT and tumor-bearing VA and VAK mice; however, CD8α^+^ γδ T cells were almost absent from tumors in either model (Fig. 2C). These observations showed that tumor-infiltrating Vγ7^+^ cells and other γδ T-cell subsets were rare.

How quickly γδ T cells might be excluded from tumors was investigated using a short-term model, wherein both alleles of *Apc* were simultaneously deleted in gut tissue, thereby maximally activating β-catenin signaling. The mouse intestine cannot tolerate loss of *Apc* in this way, so mice are euthanized 3 or 4 days after CRE recombinase induction. γδ T cells were quantified in villi of the SI of *Villin-Cre^ERT2^;Apc^F/F^* (VA^F/F^) mice and *Villin-Cre^ERT2^;Apc^F/F^;Kras^G12D/+^* (VA^F/F^K) mice. The number of γδ T cells was reduced by about 3-fold in VA^F/F^ and VA^F/F^K mice when compared with Cre-negative controls (Fig. 2D and E), indicating that deletion of *Apc* in epithelial cells had a rapid impact on γδ T-cell numbers, before the overt formation of a tumor.

To investigate whether our findings in mice might parallel human colon tumors, we examined samples from three human cohorts that were collected from Scotland, Norway, and Thailand. In all three cohorts, γδ T-cell densities were higher in normal adjacent tissue than tumor tissue (Fig. 2F and G), mirroring our observations in mouse models. Moreover, levels of γδ T cells were higher in the Scottish cohort when compared with the Norway and Thailand cohorts (Fig. 2G). We performed RNA-seq analysis on 82 human colon cancer samples from the Scotland cohort from which immunohistochemical γδ T-cell density data were available to glean information on the subtypes of γδ T cells present in these tumors. *TRGV4* transcripts were more abundant than *TRGV9* transcripts within the same tumor (Fig. 2H), indicating that Vγ4^+^Vδ1^+^ cells, which reflect colonic IEL, are on aggregate more abundant than Vγ9^+^Vδ2^+^ cells, which are typical of peripheral blood. These data corroborate but substantially extend findings by others (41, 42).

### *Btnl* molecules are downregulated in colorectal cancer

**Figure 3.**
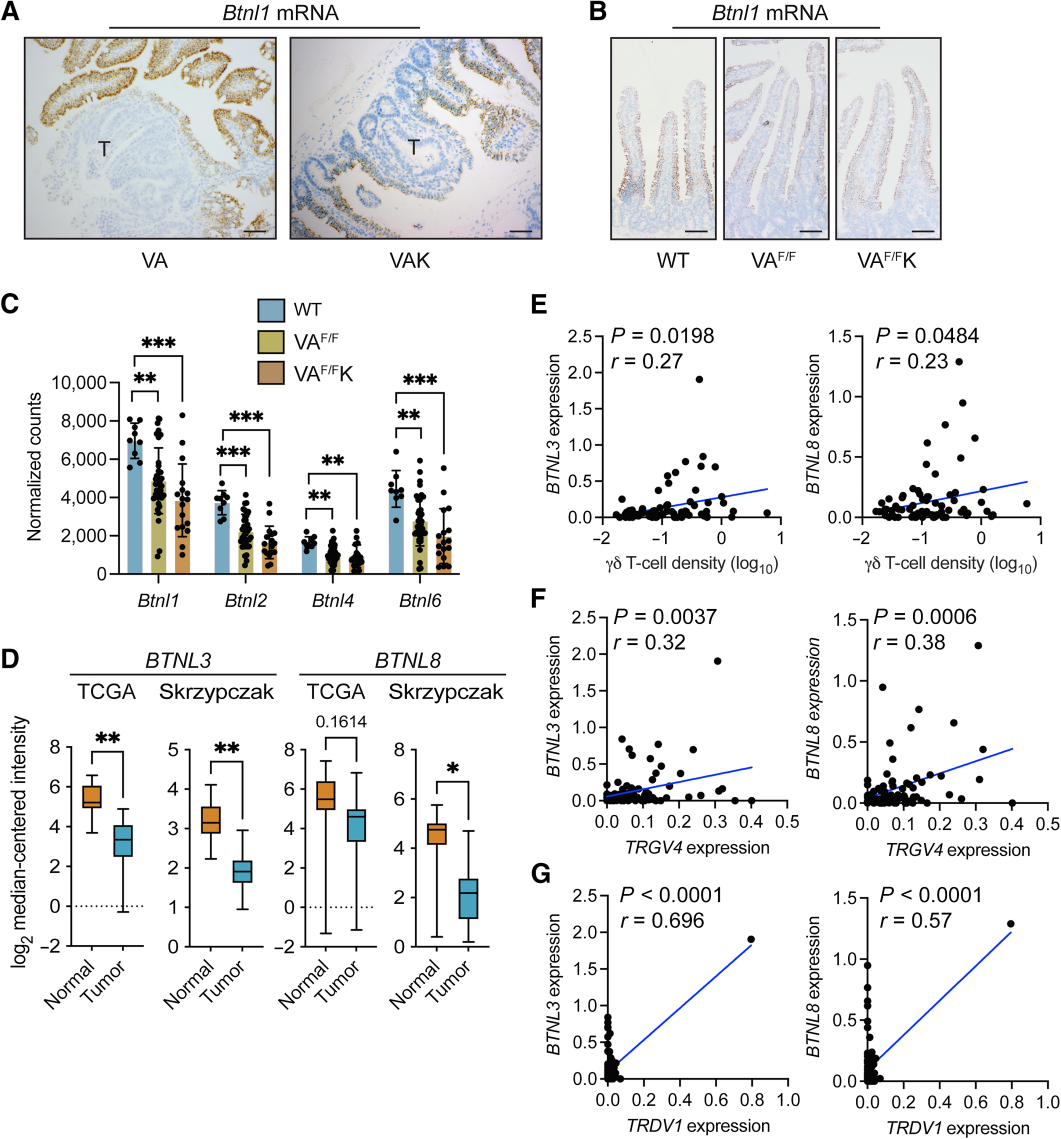
Expression of butyrophilin-like molecules is reduced in gut tumors. **A,** Images of intestinal tissue from indicated models (*n* = 4) stained for *Btnl1* mRNA. T, tumor; scale bar, 100 μm. **B,** Images of intestinal tissue from indicated models (*n* = 4) stained for *Btnl1* mRNA; scale bar, 100 μm. **C,** Butyrophilin-like mRNA expression shown by bar graph generated from RNA-seq data from WT (*n* = 9), VA^F/F^ (*n* = 36), and VA^F/F^K (*n* = 17) mice. Data are presented as mean ± SD. **D,** Expressions of *BTNL3* and *BTNL8* in normal human colonic tissue and tumor tissue from TCGA (*n* = 19 normal, 101 tumor; ref. 9) and Skrypczak (*n* = 24 normal, 45 tumor; ref. 27) datasets. Data are presented as median ± min/max. **E–G,** Correlation between indicated molecules as determined by TempO-Seq and γδ T-cell density determined by IHC in the Scotland cohort from 77 matched pairs. Units on axes are normalized read counts x 10^3^. Each dot represents one tumor. *P* and *r* values determined by Pearson correlation. *, *P* < 0.05; **, *P* < 0.01; ***, *P* < 0.001 (Mann–Whitney *U* test or one-way ANOVA followed by Tukey *post hoc* test).

We investigated the expression of *Btnl* genes, which are essential to the phenotypic maintenance of Vγ7^+^ IEL in the adult gut (4, 5, 7). When tumor sections from VA and VAK mice were stained for *Btnl1* mRNA, expression was apparent in epithelial cells surrounding adenomas but was absent from cancer cells (Fig. 3A). The kinetics of this loss of *Btnl1* expression were examined in the short-term VA^F/F^ and VA^F/F^K models. In these models, *Btnl1* expression was slightly reduced when compared with WT SI (Fig. 3B). To verify this reduction, RNA-seq data from the SI of WT, VA^F/F^ and VA^F/F^K mice were analyzed for *Btnl1*, *Btnl2, Btnl4*, and *Btnl6* gene expression (14, 15). This analysis showed reduced RNA expression of all four *Btnl* family members following deletion of *Apc* in gut tissue (Fig. 3C).

We interrogated two human gene expression datasets, The Cancer Genome Atlas (TCGA) and Skrzypczak (9, 27), to determine whether the expression levels of *BTNL3* or *BTNL8*—homologs of mouse *Btnl1* and *Btnl6*—were different between normal gut and tumor tissues. *BTNL3* expression levels were higher in normal tissue than tumor tissue in both the datasets, whereas *BTNL8* expression was only higher in normal tissue in the Skrzypczak dataset (Fig. 3D). Together, our analyses demonstrate an evolutionarily conserved reduction of *BTNL* expression in tumors across species.

The relationship between expressions of *BTNL3* and *BTNL8* and γδ T-cell infiltration in human tumors was investigated in the Scotland cohort. Gene expression values were plotted with γδ T-cell density values from matched samples. Both *BTNL3* and *BTNL8* mRNAs were positively correlated with γδ T-cell density, with human tumors exhibiting high expression of *BTNL3* and *BTNL8* containing more γδ T cells than tumors with low levels of *BTNL3* and *BTNL8* (Fig. 3E). To more specifically address the relationship between Vγ4^+^Vδ1^+^ IELs, and *BTNL3* and *BTNL8* levels, we compared *TRGV4* and *TRDV1* expression levels with *BTNL3/8* expression levels. *TRGV4* mRNA was positively correlated with both *BTNL3* and *BTNL8* expression (Fig. 3F). *TRDV1* mRNA was also positively correlated with both *BTNL3* and *BTNL8* expressions, although, *TRDV1* mRNA was not detected in 33 of 82 samples (Fig. 3G). These data support the notion that loss of *BTNL* molecules in tumors is directly associated with the loss of Vγ4^+^Vδ1^+^ IELs in the tumor microenvironment of human tumors.

### β-catenin signaling negatively regulates *Btnl* expression

**Figure 4.**
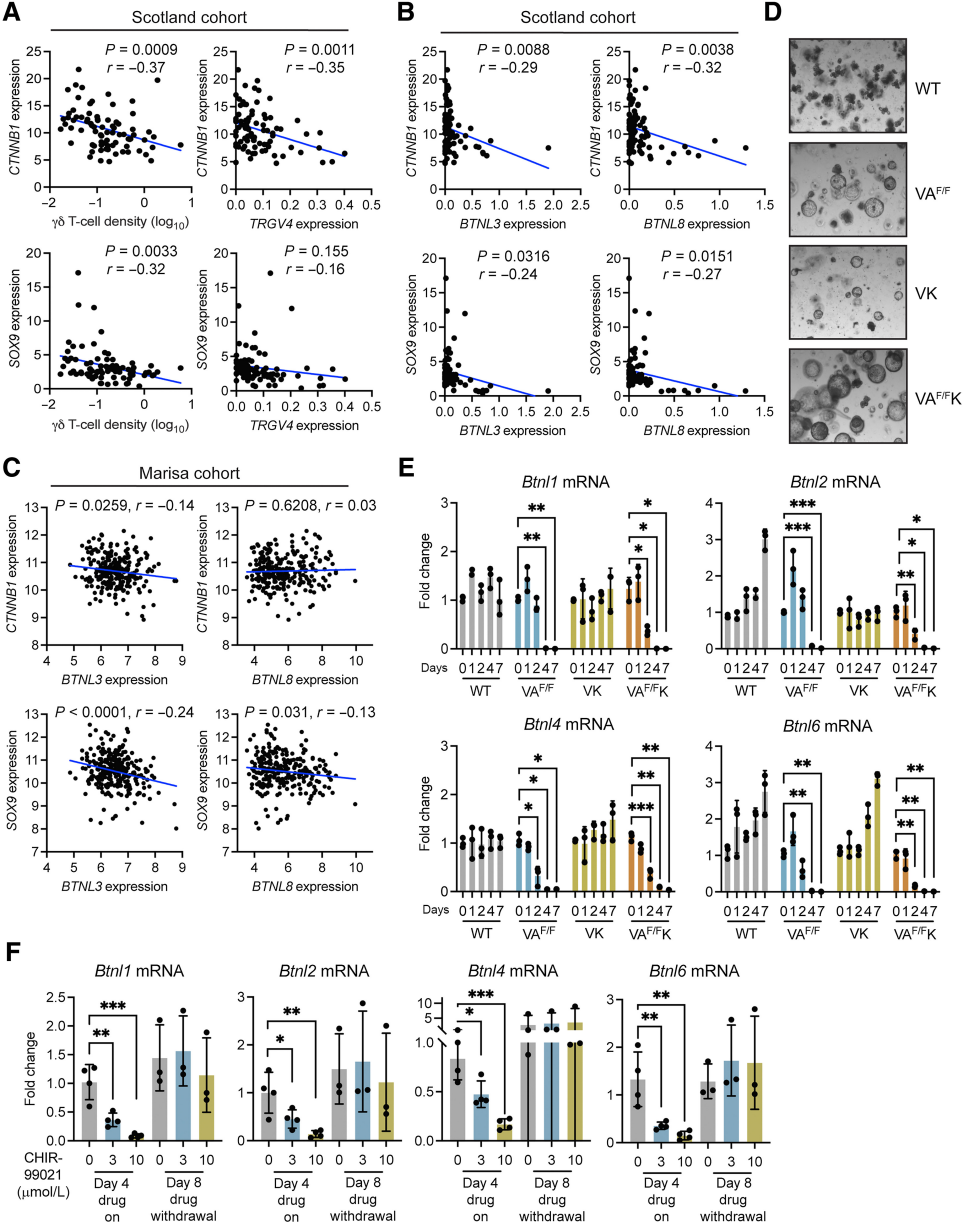
Activation of β-catenin decreases butyrophilin-like molecule expression. **A** and **B,** Correlation between indicated genes as determined by TempO-seq and γδ T-cell density determined by IHC in the Scotland cohort. Units on axes are normalized read counts × 10^3^. Each dot represents one tumor (*n* = 77 left and 82 right). *P* and *r* values determined by Pearson correlation. **C,** Correlation between *CTNNB1* or *SOX9* expression and *BTNL3* or *BTNL8* expression in the Marisa cohort (28). Units on axes are normalized counts × 10^3^. Each dot represents one tumor (*n* = 258). *P* and *r* values determined by Pearson correlation. **D,** Images of organoids derived from indicated mouse models taken 4 days after tamoxifen treatment. **E,** Fold change in expression levels of indicated genes in organoids from various genotypes measured at indicated days after tamoxifen treatment. Each dot represents one organoid derived from one mouse (*n* = 3). **F,** Fold change in expression levels of indicated genes in WT organoids treated with 3 or 10 μmol/L CHIR-99021 for indicated days. Each dot represents one organoid derived from one mouse (*n* = 3). Data are presented as mean ± SD. *, *P* < 0.05; **, *P* < 0.01; ***, *P* < 0.001 (one-way ANOVA followed by Dunnett *post hoc* test).

Prompted by the mouse data, we explored the relationship between WNT signaling, loss of *BTNL* molecules in cancer cells, and γδ T-cell exclusion in human tumors. Expression levels of *CTNNB1* (β-catenin) and *SOX9*, which is a transcriptional target of the β-catenin transcription factor complex (43), were plotted together with γδ T-cell density values determined by IHC from the same, matched tumor samples from the Scotland cohort. This analysis revealed that higher expression levels of *CTNNB1* and *SOX9* were correlated with low numbers of γδ T cells in human colon cancer (Fig. 4A). Similarly, *TRGV4* mRNA negatively correlated with both *CTNNB1* and *SOX9* expressions, although this correlation did not reach significance for the *SOX9* comparison (Fig. 4A). We did not explore correlations with *TRDV1* mRNA owing to the absence of detectable expression levels in many samples. In these human tumors, high *CTNNB1* and *SOX9* expression levels were correlated with low *BTNL3* and *BTNL8* expressions (Fig. 4B). To validate these findings, we analyzed the Marisa cohort, a publicly available microarray dataset containing 258 human colon cancer samples (28). Within this dataset, high *CTNNB1* and *SOX9* expression levels correlated with low *BTNL3* expression levels, whereas *SOX9* also negatively correlated with *BTNL8* expression (Fig. 4C). A correlation between WNT surrogate genes and other butyrophilin family members, namely *BTN3A1* and *BTN2A1*, which bind Vγ9Vδ2^+^ cells, was not observed in the Scotland cohort dataset, except for *CTNNB1* and *BTN2A1* expression levels (Supplementary Fig. S1A). These data support our hypothesis of a unique relationship between WNT signaling, Vγ4^+^Vδ1^+^ IEL exclusion from tumors, and a loss of *BTNL* genes in cancer cells.

To explore a mechanistic link between WNT signaling activation and downregulation of *Btnl* gene expression, we developed an *ex vivo* transformation assay using intestinal organoids derived from tamoxifen-naïve WT, VA^F/F^, VK, and VA^F/F^K mice. Cells were treated with tamoxifen *in vitro* to induce deletion of *Apc* or expression of mutant KRAS via Cre recombinase. Tamoxifen treatment failed to influence the shape or size of organoids derived from WT mice (Fig. 4D). By contrast, tamoxifen altered the morphology of organoids harboring *Apc* and *Kras^G12D^* alleles, transforming their normal, budding shape into large spheres typical of tumor-derived organoids (Fig. 4D). Gene expression was measured in these four groups of organoids over the course of 1 week after tamoxifen treatment. We found that WNT pathway target genes, including *Lgr5, Sox9, Axin2*, and *Cd44*, were upregulated in VA^F/F^ organoids, but not in WT and VK organoids. *Lgr5* and *Cd44* were upregulated in VA^F/F^K organoids (Supplementary Fig. S2A). These results show that the organoid system recapitulated cancer cell transformation *in vivo* by β-catenin signaling. Expression of *Btnl1, Btnl2, Btnl4*, and *Btnl6* mRNA was measured in these four groups of organoids (Fig. 4E). Whereas expression of these genes remained constant in WT organoids, the deletion of *Apc* resulted by day 4 in reduced expression of all *Btnl* RNAs assayed. Activation of mutant KRAS (VK) had no effect on *Btnl* expression. However, the combination of *Apc* deletion and mutant KRAS expression in VA^F/F^K organoids accelerated *Btnl* downregulation with reduced expression apparent by day 2 (Fig. 4E). These observations demonstrate that β-catenin activation *via* loss of *Apc* negatively regulates *Btnl* gene expression.

As an alternative approach to genetic manipulation of WNT signaling, organoids from WT mice were treated with the GSK3β inhibitor CHIR-99021 to activate β-catenin. Expression of *Btnl1, Btnl2, Btnl4*, and *Btnl6* mRNA was measured after four days of treatment using two different concentrations of CHIR-99021. CHIR-99021 treatment reduced expression of all *Btnl* mRNAs assayed when compared with controls in a dose-dependent manner (Fig. 4F), thus supporting the hypothesis that activated β-catenin downregulates *Btnl gene* expression. The reversibility of this effect was tested by treating WT organoids with CHIR-99021 for 4 days, washing off drug, then culturing the treated organoids for another 4 days without drug. On day 8 after treatment began, expressions of *Btnl1, Btnl2, Btnl4* and *Btnl6* mRNA were measured by qPCR. Withdrawal of CHIR-99021 restored *Btnl1, Btnl2, Btnl4*, and *Btnl6* mRNA expression to baseline or higher levels in these organoids (Fig. 4F).

### *Btnl* genes are regulated by HNF4 transcription factors

**Figure 5.**
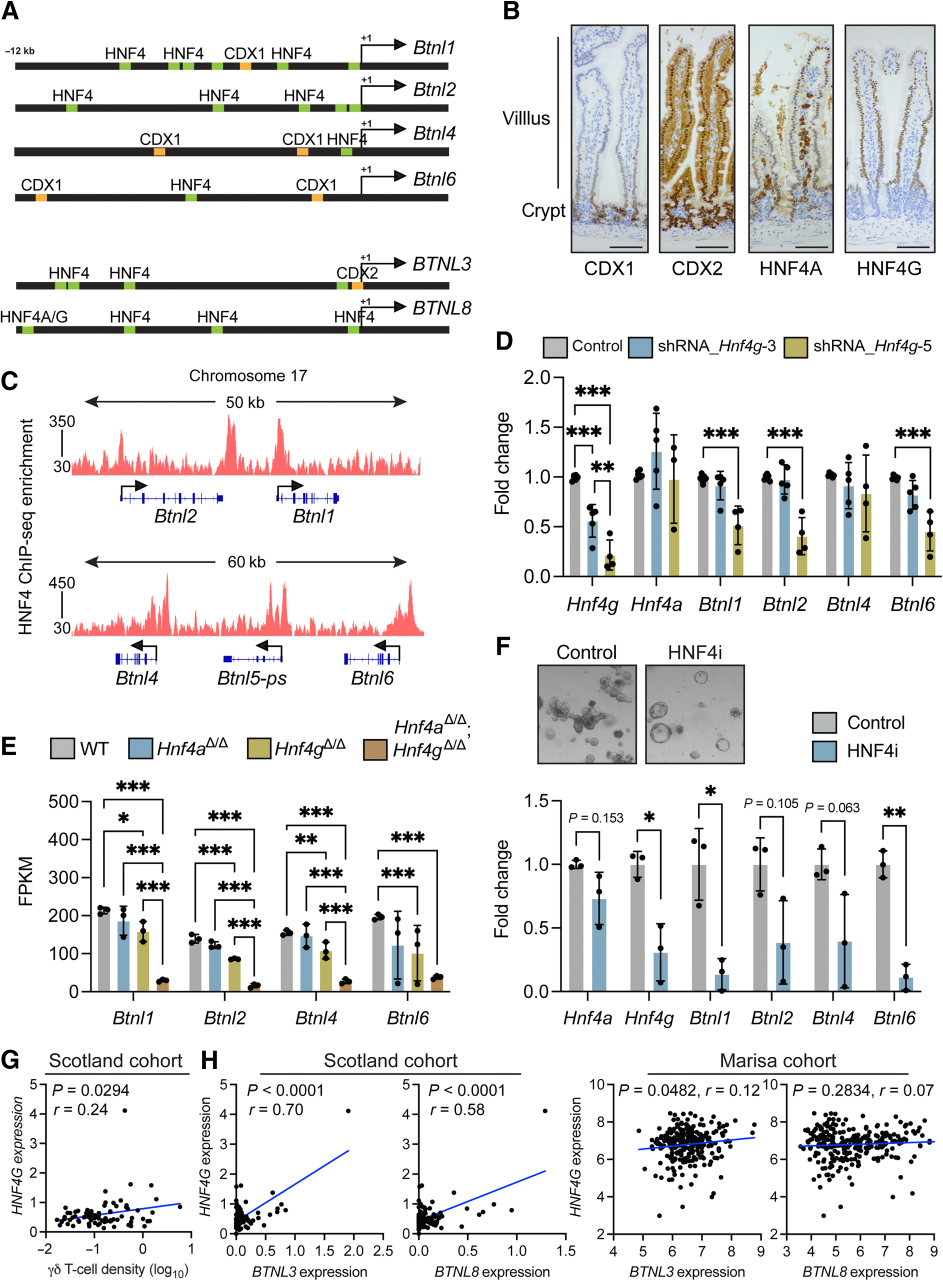
HNF4A and HNF4G regulate butyrophilin-like molecule expression in normal gut tissue. **A,** Schematic of mouse and human promoter regions of indicated genes. Putative HNF4A/G-binding sites are shown in green; CDX1 and CDX2 binding sites are shown in orange. **B,** Images of CDX1, CDX2, HNF4A and HNF4G protein expressions in SI of WT mice (*n* = 4); scale bar, 500 μm. **C,** Integrative Genomics Viewer analysis of HNF4A/HNF4G ChIP-seq data at mouse *Btnl* gene loci. **D,** Fold change in expression levels of indicated genes in WT organoids transduced with shRNA constructs targeting *Hnf4 g* transcripts. Each dot represents one organoid from one mouse (*n* = 4). Data are presented as mean ± SD. **E,** Butyrophilin-like molecule expression determined by RNA-seq analysis of SI in indicated mouse models. Each dot represents one mouse (*n* = 3). Data are presented as mean ± SD. **F,** Images of organoids from WT mice treated with DMSO control or HNF4A/G inhibitor (HNF4i) representative of 3/group. Fold change in expression levels of indicated genes. Each dot represents one organoid from one mouse (*n* = 3). Data are presented as mean ± SD. **G,** Correlation between *HNF4G* expression as determined by TempO-seq and γδ T-cell density determined by IHC in the Scotland cohort. Units on axes are normalized counts x 10^3^. Each dot represents one tumor (*n* = 77). *P* and *r* values determined by Pearson correlation. **H,** Correlation between *BTNL3* or *BTNL8* expression and *HNF4G* expression units on axes are normalized counts x 10^3^. Each dot represents one tumor (*n* = 82 Scotland cohort, 258 Marisa cohort). *P* and *r* values determined by Pearson correlation. *, *P* < 0.05; **, *P* < 0.01; ***, *P* < 0.001 (unpaired *t* test or one-way ANOVA followed by Tukey *post hoc* test).

To understand how WNT signaling negatively affects *Btnl* gene expression, we investigated how *Btnl* molecules are regulated in normal tissue. We searched for potential transcription factor–binding sites in the promoter regions of these genes. Using a publicly available database (OregAnno), we generated a list a putative transcription factor–binding sites, then narrowed down the list by focusing on gut-specific transcription factors. This analysis uncovered two sets of paralogs, CDX1 and CDX2, and HNF4A and HNF4G, with multiple binding sites found within 12 kb upstream of mouse and human *BTNL* gene start sites (Fig. 5A).

We determined whether CDX1, CDX2, HNF4A or HNF4G were localized specifically to the villus where BTNL molecules are expressed. CDX1 was expressed in crypt regions and lower villus, but expression decreased as enterocytes moved up the villus (Fig. 5B). CDX2 was expressed in both crypts and villi with higher expression in the crypt. HNF4A was also expressed in both crypts and villi; staining was also observed in cells residing within the lamina propria. HNF4G expression was specific to enterocytes in the villus, as no expression was observed in crypt regions (Fig. 5B). These data suggested HNF4G as the prime candidate for *Btnl* gene regulation, given their overlapping patterns of expression in the villus. However, all four transcription factors are expressed in the villus to some extent.

We investigated whether CDX1 and CDX2 mediated *Btnl1, Btnl2, Btnl4*, and *Btnl6* transcription. Organoids from WT mice were transduced with 5 shRNA constructs targeting *Cdx1* or *Cdx2* mRNA. Two constructs achieved good knockdown efficiency for *Cdx1*, although organoid morphology and expression of *Btnl* molecules remained unchanged (Supplementary Fig. S3A and S3B). Attempts to knockdown *Cdx2* proved difficult as organoids transduced with these constructs often died. In two replicate experiments where organoids survived antibiotic selection, knockdown of *Cdx2* was sufficiently achieved with the shRNA_*Cdx2*–2 construct, but this failed to impact on organoid morphology or *Btnl* gene expression (Supplementary Fig. S3C and S3D). These data suggested that CDX2 was required for organoid survival. Indeed, conditional deletion of *Cdx2* in adult intestine is lethal (44). We concluded from these experiments that CDX1 and CDX2 were not required specifically for *Btnl1, Btnl2, Btnl4*, and *Btnl6* transcription.

HNF4A and HNF4G are paralogs that bind fatty acids and whose functions are somewhat redundant (23, 45, 46). These transcription factors recognize a nearly identical consensus motif on DNA, and they exhibit 98.7% commonality in DNA-binding profiles (23). It should be noted that HNF4A is expressed outside the gut at sites such as liver (47), where BTNL molecules are not expressed (4). To determine whether HNF4A and HNF4G bound the promoter region of *Btnl1, Btnl2, Btnl4*, and *Btnl6* genes, we analyzed chromosome 17 in a HNF4 ChIP-seq dataset from mouse SI (23). This analysis confirmed that HNF4A/G bound all *Btnl* gene promoter regions (Fig. 5C).

We investigated whether HNF4A and HNF4G activity was causally linked to *Btnl* expression. Organoids from WT mice were transduced with 5 shRNA constructs targeting *Hnf4a* or *Hnf4g* mRNA. Organoid morphology was unaffected by *Hnf4a* constructs (Supplementary Fig. S3E). Knockdown of *Hnf4a* was not successful. Instead of reduced expression, we observed higher expressions of *Hnf4a* and *Hnf4g* in these cells, concomitant with higher expression of *Btnl1, Btnl2, Btnl4*, and *Btnl6* genes (Supplementary Fig. S3F). These findings suggested that a feedback mechanism may be active, preventing *Hnf4a* knockdown, but provided indirect evidence that increased HNF4A and HNF4G expressions correlated with increased *Btnl* expression. To clarify this situation, we transduced MODE-K enterocytes that do not express *Hnf4a* with a series of cDNAs encoding gut-associated transcription factors, including *Cdx1*, *Cdx2*, and *Hnf4a*. Only in *Hnf4*-transduced cells was there overt upregulation of *Btnl* mRNAs, specifically those for *Btnl4* and *Btnl6* (Supplementary Fig. S3G), whereas *Cdx1, Cdx2, Creb3l3, Gata5*, and *Isx* failed to influence *Btnl* gene expression. For *Hnf4g* targeting in organoids from WT mice, two constructs achieved good knockdown efficiency with the shRNA_*Hnf4g-5* construct exhibiting superior efficiency. However, only the shRNA_*Hnf4g-5* construct reduced expression of *Btnl1*, *Btnl2*, and *Btnl6* without affecting expression of *Btnl4* (Fig. 5D). This was accompanied by the occasional appearance of sphere-shaped organoids (Supplementary Fig. S3H), as observed in other reports (23) and indicative of a stem cell–like state. Collectively, our data demonstrate that HNF4 transcription factors are regulators of *Btnl* gene expression, although there are seemingly differences in the degrees to which specific *Btnl* genes are dependent upon or influenced by HNF4A and HNF4G, respectively. These data further integrate *Btnl* expression with physiological enterocyte differentiation (23).

We analyzed *Btnl* gene expression in mouse models deficient for HNF4A or HNF4G or both. An RNA-seq dataset derived from intestine of WT, *Villin-Cre^ERT2^;Hnf4a^F/F^* (*Hnf4a^Δ/Δ^*) mice, *Hnf4g^—/—^* (*Hnf4g^Δ/Δ^*) mice and *Hnf4a^Δ/Δ^;Hnf4g^Δ/Δ^* mice was used for this purpose (23). In these mice, deletion of *Hnf4a* failed to alter *Btnl* gene expression, whereas deletion of *Hnf4 g* reduced expression of all four *Btnl* genes (Fig. 5E). Simultaneous deletion of *Hnf4a* and *Hnf4 g* led to the most pronounced loss of *Btnl* expression when compared with WT tissue. *Btnl1, Btnl2*, and *Btnl4* (but not *Btnl6*) mRNA was also lower in *Hnf4a^Δ/Δ^;Hnf4g^Δ/Δ^* intestine than in *Hnf4a^Δ/Δ^* or *Hnf4g^Δ/Δ^* intestine (Fig. 5E). We then used an inhibitor that targets both HNF4A and HNF4G, BI-6015 (HNF4i), in organoids from WT mice. This drug altered the morphology of the organoids, transforming them into spheres (Fig. 5F), similar to the morphology of *Hnf4a^Δ/Δ^;Hnf4g^Δ/Δ^* organoids previously described (23). Inhibition of these transcription factors by BI-6015 reduced expression of *Hnf4a* and *Hnf4g*, as well as *Btnl1, Btnl2, Btnl4*, and *Btnl6* mRNA (Fig. 5F). Together, these data demonstrate that HNF4G is the main regulator of *Btnl* molecule expression with cooperation from HNF4A in enterocytes.

The relationship between transcription factor expression, γδ T-cell infiltration and *BTNL* gene expression was examined in human tumors. In the Scotland cohort, there was no correlation between *CDX1, CDX2*, and *HNF4A* expressions and γδ T-cell density (Supplementary Fig. S4A–S4C). There was a positive correlation between *CDX1*, *BTNL3*, and *BTNL8* expressions in both the Scotland and Marisa cohorts (Supplementary Fig. S4D), but correlations between *CDX2* or *HNF4A* and *BTNL3* or *BTNL8* were absent or inconsistent among both cohorts (Supplementary Fig. S4E and S4F). In contrast with the other transcription factors, increased *HNF4G* expression was correlated with higher γδ T-cell density in human tumors (Fig. 5G). Increased *HNF4G* expression also correlated with increased *BTNL3* expression in both the Scotland and Marisa cohorts, whereas the relationship with *BTNL8* expression was only observed in the Scotland cohort (Fig. 5H). These data establish an association between *HNF4G*, *BTNL* expressions, and tumor-infiltrating γδ T cells in human tumors and point to HNF4G regulation of *BTNL* gene expression as being conserved across species.

### Increased WNT signaling disrupts HNF4 expression, *Btnl* expression and γδIELs

We hypothesized that disruption of the WNT gradient in the mouse intestine would interfere with enterocyte-specific HNF4G and *Btnl* gene expression and subsequently γδIEL abundance. To test this hypothesis, we used *Vil1-Grem1* mice in which *Gremlin1* is under the control of the *Vil1* promoter. These mice develop ectopic crypts in the villi due to the antagonist actions of GREM1 on bone morphogenic proteins, a consequence of which is increased WNT signaling in the villi (13). Nuclear SOX9 was used to identify ectopic crypts in the villi of *Vil1-Grem1* mice (Supplementary Fig. S5A). These SOX9-high, WNT-high ectopic crypts maintained HNF4A as in normal crypts, but lost expressions of HNF4G and *Btnl1* mRNA (Supplementary Fig. S5A). Moreover, we quantified γδ T cells in villi of *Vil1-Grem1* mice and found that these cells were reduced when compared with WT mice (Supplementary Fig. S5A and S5B). These results show that WNT signaling suppresses the HNF4G–*Btnl*–Vγ7^+^ cell axis.

Further testing of our hypothesis was carried out in an additional model that is WNT-ligand dependent in which R-spondin 3 (RSPO3) is expressed from LGR5^+^ stem cells: *Lgr5-Cre^ERT2^;Rspo3^INV^* mice (12). In this model, increased WNT signaling induces greater numbers of crypt regions, as demonstrated by increased SOX9^+^ cells at the base of the intestine, and reduced villus length (Supplementary Fig. S5C). We investigated HNF4A, HNF4G, and *Btnl1* expressions in these mice. Staining patterns of these molecules were consistent with expression in intestine from WT mice where HNF4A was expressed in crypt regions and enterocytes, whereas HNF4G and *Btnl1* expressions were restricted to enterocytes (Supplementary Fig. S5C). However, the expansion of WNT-high crypt regions and reduced villus length resulted in fewer γδ T cells in the villi of these in *Lgr5-Cre^ERT2^;Rspo3^INV^* mice, when compared with Cre-negative mice (Supplementary Fig. S5C and S5D). To determine whether reduced γδ T-cell numbers could be restored by interference with WNT signaling, *Lgr5-Cre^ERT2^;Rspo3^INV^* mice were treated with the porcupine inhibitor (PCPNi) LGK-974 to block the secretion of WNT ligands and prevent its activation of β-catenin. Expression patterns of HNF4A in crypt and villi regions as well as HNF4G and *Btnl1* mRNA in enterocytes were unaltered by LGK-974 treatment. However, γδ T-cell numbers in the villi of these mice increased (Supplementary Fig. S5C and S5D). These data provide evidence that aberrant β-catenin activation in normal intestinal tissue disrupts γδIEL abundance.

### *Hnf4a* and *Hnf4g* are suppressed by WNT signaling during tumor initiation

**Figure 6.**
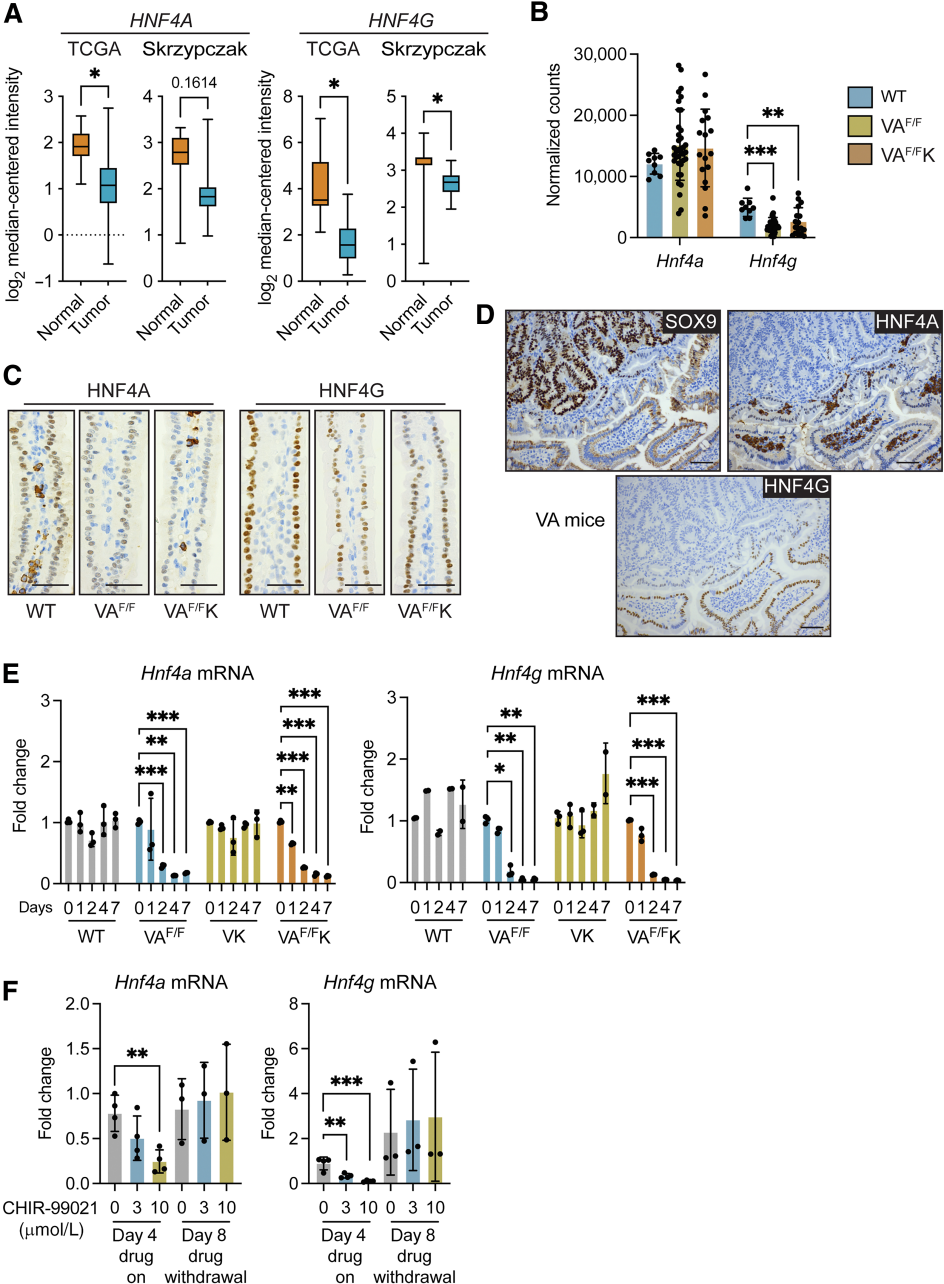
Activation of β-catenin decreases *Hnf4a and Hnf4g* expressions. **A,** Expressions of *HNF4A* and *HNF4G* in normal human colonic tissue and tumor tissue from TCGA (*n* = 19 normal, 101 tumor) and Skrypczak (*n* = 24 normal, 45 tumor) datasets. Data are presented as median ± minimum/maximum. *, *P* < 0.05 (Mann–Whitney *U* test). **B,***Hnf4a* and *Hnf4g* expressions are determined by RNA-seq analysis of SI in WT (*n* = 9), VA^F/F^ (*n* = 36), and VA^F/F^K (*n* = 17) mice. Each dot represents one mouse. **C,** Images of HNF4A and HNF4G protein expressions in SI of indicated models (*n* = 4); scale bar, 500 μm. **D,** Images of intestinal tissue from tumor-bearing VA mice stained for indicated proteins; scale bar, 500 μm. **E,** Fold change in expression levels of *Hnf4a* and *Hnf4g* in organoids from various genotypes measured at indicated days after tamoxifen treatment. Each dot represents one organoid from one mouse (*n* = 3). **F,** Fold change in expression levels of *Hnf4a* and *Hnf4g* in WT organoids treated with 3 or 10 μmol/L CHIR-99021 for indicated days. Each dot represents one organoid from one mouse (*n* = 3). Data are presented as mean ± SD. **, *P* < 0.01; ***, *P* < 0.001 (one-way ANOVA followed by Dunnett *post hoc* test).

We compared *HNF4A* and *HNF4G* expressions between normal human colon and tumor tissues in the TCGA and Skrzypczak datasets. Both *HNF4A* and *HNF4G* were reduced in tumor tissue from both datasets (Fig. 6A), mirroring reduced *BTNL3* and *BTNL8* expressions in human tumors (Fig. 3D).

We investigated whether expressions of *Hnf4a* and *Hnf4 g* mRNAs were affected by WNT signaling by examining mRNA levels in the SI of WT, VA^F/F^ and VA^F/F^K mice. This analysis showed that *Hnf4a* levels were similar between normal Sl and *Apc*-deficient SI tissues, whereas *Hnf4 g* levels were reduced in *Apc*-deficient tissue (Fig. 6B). IHC on these models revealed that nuclear staining of both HNF4A and HNF4G was reduced or even absent from epithelial cells in the villus of VA^F/F^ and VA^F/F^K tissue when compared with WT tissue (Fig. 6C). The addition of mutant KRAS to *Apc* loss had no influence over decreased expression of HNF4A and HNF4G. The discrepancy between *Hnf4a* mRNA and HNF4A protein levels may be explained by expression of HNF4A^+^ stromal cells in the lamina propria. These data show that expressions of HNF4A and HNF4G are rapidly reduced or lost completely in cells with high β-catenin activity.

End-stage tumors from VA mice were evaluated for the presence of HNF4A and HNF4G. Nuclear SOX9 staining was used to identify WNT-high tumors. We found that HNF4A and HNF4G were completely absent from cancer cells, whereas normal adjacent epithelial cells maintained nuclear HNF4A and HNF4G staining (Fig. 6D). This pattern of expression mimicked loss of *Btnl1* staining in tumors from the same mouse model (Fig. 3A).

We used the organoid transformation assay to test the kinetics of *Hnf4a* and *Hnf4g* downregulation after β-catenin activation. After tamoxifen treatment, expression of these molecules remained constant in WT organoids (Fig. 6E). The deletion of *Apc* resulted in reduced expression of *Hnf4a* and *Hnf4g* by day 2, which was 2 days earlier than was observed for *Btnl* mRNA downregulation, as shown in Fig. 4E. Induction of oncogenic KRAS had no effect on *Hnf4a* and *Hnf4g* gene expressions, but the combination of *Apc* deletion and mutant KRAS expression in VA^F/F^K organoids resulted in a downregulation of *Hnf4a* and *Hnf4g* by day 1 or 2 (Fig. 6E). These observations indicated that suppression of *Hnf4a* and *Hnf4g* RNAs by β-catenin preceded the downregulation of *Btnl* gene expression. Treatment of WT organoids with the GSK3β inhibitor CHIR-99021 reduced expression of *Hnf4a* and *Hnf4g* (Fig. 6F). As observed with *Btnl1, Btnl2, Btnl4*, and *Btnl6* expressions (Fig. 4F), the inhibition of *Hnf4a* and *Hnf4g* mRNA was reversible after withdrawal of CHIR-99021 with expression levels returning to normal on day 8 (Fig. 6F).

Analysis of CUT&RUN datasets for the WNT/β-catenin mediator LEF1 in human HEK293T cells stimulated with 10 μmol/L CHIR-99021 (34) revealed a LEF1-binding site downstream of the *HNF4G* locus (orange track in Supplementary Fig. S6A). Using an ATAC-seq dataset (35), we found that this LEF1-bound genomic region had an open chromatin signal in response to CHIR-99021 treatment with high evolutionary conservation (Supplementary Fig. S6B), pointing to an active regulatory site. The presence of JASPAR-predicted LEF1 consensus sequences, which match the binding profile, further supported the notion that this genomic locus is a *bona fide* WNT Responsive Element (WRE; Supplementary Fig. S6C). Finally, GREAT unbiasedly annotated this WRE to the *HNF4G* promoter (Supplementary Fig. S6D), suggesting its direct regulation of *HNF4G*. By contrast, no obvious signal was detected for LEF1 binding in proximity to the *HNF4A* locus (Supplementary Fig. S6E).

Together, these data are consistent with the notion that β-catenin suppresses *Btnl1/2/4/6* gene expression, via downregulation of HNF4A and HNF4G.

### Ectopic expression of BTNL1/BTNL6 in colon cancer cells fails to impact tumor progression or γδ T-cell infiltration

We investigated whether introduction of the BTNL1/BTNL6 heterodimer in BTNL1/BTNL6-deficient cells was sufficient to enhance Vγ7^+^ cell–mediated cancer cell death or to induce Vγ7^+^ cell infiltration into tumors and slow tumor growth. We cloned *Btnl1* and *Btnl6* into doxycycline-inducible vectors and transduced CT26 cells with them as CT26 cells naturally lack expression of *Btnl1* and *Btnl6* mRNA (Supplementary Fig. S7A). We confirmed increased expression of *Btnl1* and *Btnl6* mRNA by doxycycline in these cells (CT26-B1/6 cells) grown *in vitro* (Supplementary Fig. S7A). We tested whether the expression of BTNL1 and BTNL6 modulated the growth pattern of these cells. Upregulation of *Btnl1* and *Btnl6* mRNA failed to influence the proliferation of CT26 cells *in vitro* (Supplementary Fig. S7B). These cells were transplanted into the flank of syngeneic mice, but the growth of tumors remained unchanged when compared with control CT26 tumors despite achieving a high induction of *Btnl1* and *Btnl6* mRNA in CT26-B1/6 tumors (Supplementary Fig. S7C and S7D). These data indicate that ectopic expression of the BTNL1/BTNL6 heterodimer had no impact on cancer cell growth.

**Figure 7.**
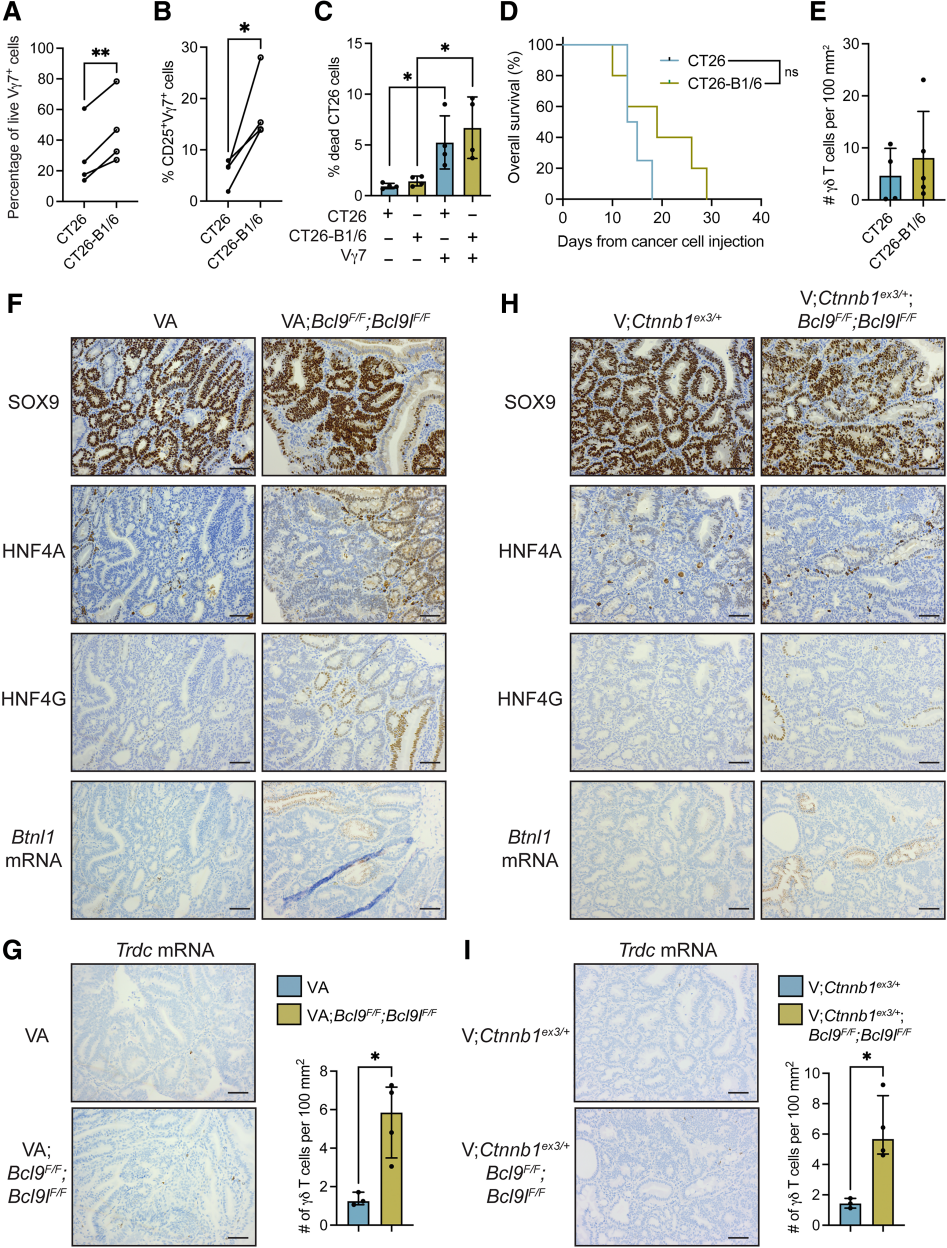
Inhibition of β-catenin transcriptional activity increases expression of HNF4A, HNF4G, and butyrophilin-like molecules. **A,** Vγ7^+^ cell viability in cocultures with CT26 or CT26-B1/6 cells. Each dot represents one paired biological replicate (*n* = 4). **B,** CD25 expression by Vγ7^+^ cells in cocultures with CT26 or CT26-B1/6 cells. Each dot represents one paired biological replicate (*n* = 4). **C,** CT26 and CT26-B1/6 cancer cell death using flow cytometry after coculture with Vγ7^+^ cell as indicated. Each dot represents one biological replicate (*n* = 4). **D,** Kaplan–Meier survival analysis of doxycycline-treated CT26 and CT26-B1/6 tumor-bearing mice (*n* = 4 CT26, 5 CT26-B1/6) using the log-rank test. **E,** γδ T-cell numbers in tumors from doxycycline-treated CT26 and CT26-B1/6 tumor-bearing mice. Each dot represents one mouse (*n* = 4 CT26, 5 CT26-B1/6). **F** and **H,** Images taken from serially stained sections of indicated protein in tumors from indicated mouse models (*n* = 3–4; scale bar, 500 μm. **G** and **I,** Images of *Trdc* expression in tumors from indicated mouse models; scale bar, 500 μm. γδ T-cell numbers in tumors. Each dot represents one mouse. Data are presented as mean ± SD. *, *P* < 0.05; **, *P* < 0.01 (paired *t* test or unpaired *t* test or one-way ANOVA followed by Tukey *post hoc* test).

Vγ7^+^ cells were cocultured with CT26 vector control cells or CT26-B1/6 cells. We observed increased viability and expression of the activation marker CD25 by Vγ7^+^ cells when cocultured with CT26-B1/6 cells as compared with control cells (Fig. 7A and B; Supplementary Fig. S7E and S7F). These data showing increased activation of γδIELs after Vγ7-BTNL1/BTNL6 interaction accord well with previous studies (5, 7). We measured death of CT26 cells in these cocultures. We found that Vγ7^+^ cells increased CT26 cell death by approximately 5-fold; however, engagement with the BTNL1/BTNL6 heterodimer had no impact on cancer cell killing by Vγ7^+^ cells (Fig. 7C; Supplementary Fig. S7G). Therefore, the ability of Vγ7^+^ cells to induce cancer cell death is independent of the BTNL1/BTNL6 heterodimer, although the BTNL1/BTNL6 heterodimer can support Vγ7^+^ cell survival and activation.

We transplanted control and CT26-B1/6 cells into the colonic submucosa of syngeneic mice by colonoscopy-guided injection and treated tumor-bearing mice with doxycycline. We confirmed increased expression of *Btnl1* mRNA by doxycycline in these tumors (Supplementary Fig. S7H). We found no difference in survival of tumor-bearing mice when *Btnl1* and *Btnl6* mRNA were induced (Fig. 7D). Moreover, the number of CD3^+^ T, CD8^+^ T, and γδ T was the same between groups (Fig. 7E; Supplementary Fig. S7I–S7K). Therefore, we concluded that the BTNL1/BTNL6 heterodimer may support Vγ7^+^ cell survival in tumors, but BTNL1/BTNL6 are not sufficient to induce Vγ7^+^ cell infiltration into the tumor microenvironment (or even other T cells), suggesting that other molecules are necessary to attract Vγ7^+^ cells into tumors.

### The HNF4–BTNL–γδ T-cell axis is restored in tumors by interference with β-catenin activity

Because ectopic expression of BTNL1/BTNL6 was insufficient to mediate recruitment of γδ T cells, we asked whether inhibition of β-catenin signaling could reverse γδ T-cell exclusion from tumors. The β-catenin transcription factor complex consists of several components, including B-cell lymphoma 9 (BCL9) and BCL9-like (BCL9L; refs. 48, 49), whose deletion in mouse tumor models abrogates β-catenin–mediated transcription (14, 50). Therefore, we investigated expression levels of *Hnf4a, Hnf4g, Btnl1, Btnl2, Btnl4*, and *Btnl6* in tissue where *Apc* is deleted and *Bcl9* and *Bcl9*L are absent. For this purpose, we examined RNA-seq data from intestinal tissue of VA^F/F^ mice and VA^F/F^;*Bcl9^F/F^;Bcl9l^F/F^* mice that were treated with tamoxifen for 4 days to induce Cre recombinase. This analysis showed that *Hnf4g*, *Btnl2*, and *Btnl4* levels were higher in VA^F/F^;*Bcl9^F/F^;Bcl9l^F/F^* intestinal tissue, whereas *Hnf4a* and *Btnl1* mRNA remained unchanged (Supplementary Fig. S7L). *Btnl6* could not be detected in this dataset.

End-stage tumors from VA and VA;*Bcl9^F/F^;Bcl9l^F/F^* mice were assessed for expression of HNF4A, HNF4G and *Btnl1* mRNA. SOX9 was used to detect WNT-high cancer cells. Expressions of HNF4A, HNF4G, and *Btnl1* mRNA were absent from tumors in VA mice (Fig. 7F). By contrast, nuclear expressions of HNF4A and HNF4G as well as *Btnl1* mRNA were apparent in some but not all areas of tumors from VA;*Bcl9^F/F^;Bcl9l^F/F^* mice (Fig. 7F). Previous reports indicate that recombination of *Bcl9^F/F^;Bcl9l^F/F^* alleles is inefficient in these mice (14), which provides an explanation for the sporadic expression pattern of HNF4A, HNF4G, and *Btnl1* mRNA in these tumors. To determine whether the restoration of HNF4A, HNF4G, and *Btnl1* expression in tumors from VA;*Bcl9^F/F^;Bcl9l^F/F^* mice affected tumor-infiltrating γδ T cells, we quantified these cells in tumor tissue. This analysis showed that γδ T cells were more abundant in tumors from VA;*Bcl9^F/F^;Bcl9l^F/F^* mice than VA mice (Fig. 7G).

Another colon cancer model, *Villin1-Cre^ERT2^;Ctnnb1*^ex3/+^ (V;*Ctnnb1*^ex3/+^) mice, was used to validate these findings, where mutant β-catenin is used to drive tumor formation (14). Nuclear SOX9 expression was used to identify WNT-high cancer cells. In this model, nuclear HNF4A expression was evident in cancer cells (albeit weak expression), whereas HNF4G and *Btnl1* expressions were lost from tumors (Fig. 7H). V;*Ctnnb1*^ex3/+^ mice were crossed with *Bcl9^F/F^;Bcl9l^F/F^* mice. Tumors from V;*Ctnnb1*^ex3/+^;*Bcl9^F/F^;Bcl9l^F/F^* mice retained expression of HNF4A. Nuclear HNF4G and *Btnl1* mRNA expressions could be observed in overlapping regions of tumors (Fig. 7H), although staining was sporadic as in tumors from VA;*Bcl9^F/F^;Bcl9l^F/F^* mice (Fig. 7F). We quantified tumor-infiltrating γδ T cells in these mice and found that tumor-infiltrating γδ T cells are more abundant in tumors from V;*Ctnnb1*^ex3/+^;*Bcl9^F/F^;Bcl9l^F/F^* mice than V;*Ctnnb1*^ex3/+^ mice (Fig. 7I). These data indicate that inhibition of β-catenin signaling reverses suppression of HNF4A/HNF4G-driven *Btnl* gene expression and exclusion of Vγ7^+^ cells in tumors.

## Discussion

Our study shows how BTNL molecule expression is lost in mutated epithelial cells by dysfunctional WNT signaling. Our data in *Btnl1*-deficient mice suggest that cancer cells thrive in the absence of Vγ7^+^ cells, in line with recent evidence from several groups reporting on the pivotal role of human and mouse γδIELs in cancer immunosurveillance (2, 42, 51). In addition to direct cancer cell killing, γδIELs may also suppress protumorigenic inflammation, such as occurs during intestinal nutrient sensing (40), to impede cancer progression. We found that normal IECs use HNF4G (most likely dimerized with HNF4A) to induce expression of *Btnl* gene expression. These data are supported by a recent study focused on HNF4A-regulated gene expression throughout gut tissue (52), where HNF4A transcriptional activity seems to be more important in the mouse large intestine than small intestine. Although these paralogs bind the same consensus sites in promoter regions and exhibit redundant functions (23), our data suggest that HNF4G is the dominant regulator of *Btnl* molecules. Indeed, *HNF4G* expression, not *HNF4A*, correlated with *BTNL3*/*BTNL8* expression and γδ T-cell infiltration into tumors in our human colon cancer dataset.

The biological basis for evasion from γδIEL immunosurveillance shown herein revolves around WNT-driven dedifferentiation of cancer cells toward a stem cell–like state. Dysregulated WNT signaling fosters the conversion of cancer cells toward a less differentiated phenotype reminiscent of LGR5^+^ stem cells that reside in intestinal crypts. LGR5^+^ stem cells, like cancer cells, fail to express HNF4G and BTNL molecules (5), making crypt regions and tumors immune privileged sites, devoid of γδIELs. Redifferentiation of colon cancer cells could reengage γδIEL immunosurveillance, and strategies to achieve redifferentiation could benefit γδIEL-based cancer immunotherapies. These approaches could be combined with anti–PD-1 therapy to boost γδIEL killing activity, particularly in patients lacking HLA expression (51). At the same time, redifferentiation would slow the proliferative signals induced by β-catenin in cancer cells. Our data suggest that redifferentiation may be possible given that inhibition of β-catenin transcriptional activity by deletion of BCL9 and BCL9 L results in reexpression of HNF4G and BTNL molecules and increased numbers of γδ T cells in tumors. This notion is supported by data from other disease settings. Individuals with celiac disease exhibit a loss of *BTNL8* expression concomitant with a loss of Vγ4^+^Vδ1^+^ IELs, but elimination of dietary gluten can restore *BTNL8* expression (53). Together, our two studies emphasize the reversibility of *BTNL* gene expression in different disease contexts. However, expression of other molecules, cytokines, and chemokines in addition to BTNLs will likely be necessary to attract γδIELs into the tumor microenvironment, as our data show that BTNLs only support survival and activation of γδIELs. Moreover, strategies to restore γδIEL immunosurveillance will be anatomical site–specific. BTNL-responsive mouse Vγ7^+^ cells and human Vγ4^+^ cells are restricted to the gut, so reengagement of endogenous γδIELs will not be possible for liver metastasis.

## Supplementary Material

Table S1 Clinicopathological characteristics of colon cancer patient cohorts.

Figure S1 WNT expression is independent of BTN3A1 and BTN2A1

Figure S2 Deletion of Apc in organoids increases WNT target genes

Figure S3 Knockdown of Cdx1, Cdx2 and Hnf4a fails to influence organoid morphology or expression of Btnl genes

Figure S4 Correlation between gut-specific transcription factors, gamma delta T cell density and BTNL genes in human tumors

Figure S5 Disruption of WNT gradient in normal intestinal villi reduces gamma delta T cells

Figure S6 Identification of a WNT responsive element (WRE) in the HNF4G genomic locus.

Figure S7 Impact of Btnl1 and Btnl6 expression on Vgamma7+ cells and the tumor microenvironment

Supplementary Figure Legends Supplementary Figure Legends
