# Supplementary figures and images for: β-catenin drives butyrophilin-like molecule loss and γδ T-cell exclusion in colon cancer
Source: Cancer Immunol Res. Author manuscript; Available in PMC 2023 Aug 4. (PMC10398359; doi:10.1158/2326-6066.CIR-22-0644)

## Supplemental Figure 1

**A**

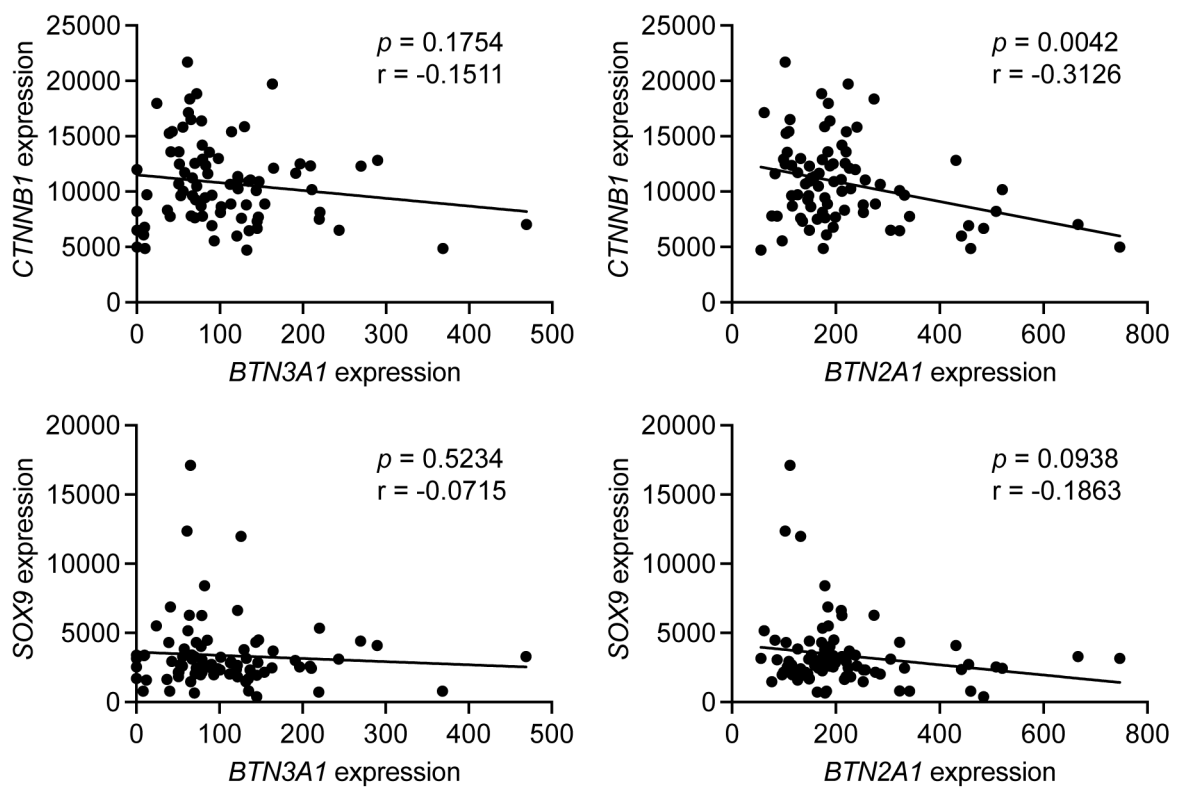

Supplement: Supplemental Figure 1 [file EMS177377-supplement-Supplemental_Figure_1.pdf]

# Supplemental Figure 2

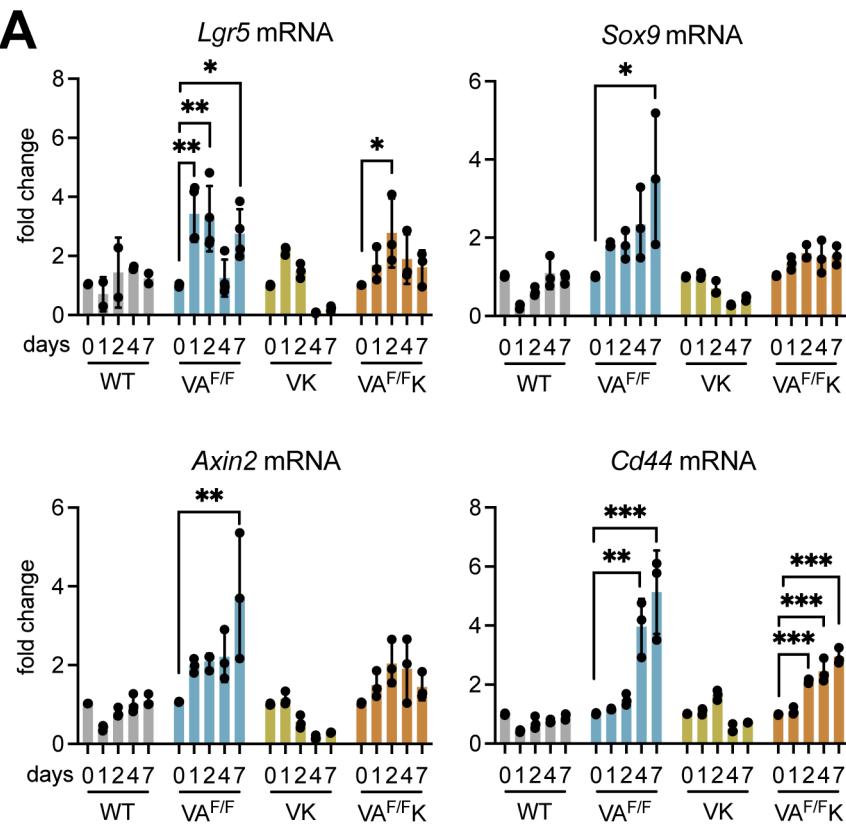

Supplement: Supplemental Figure 2 [file EMS177377-supplement-Supplemental_Figure_2.pdf]

# Supplemental Figure 3

**A**

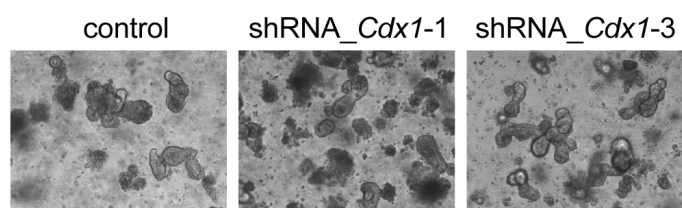

**B**

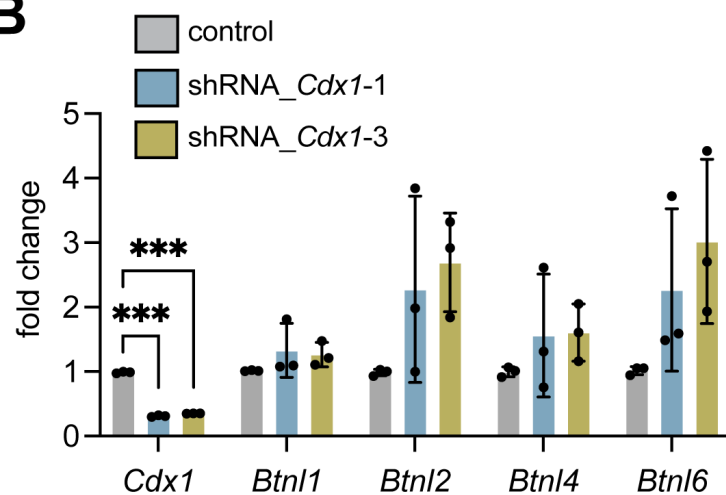

**C**

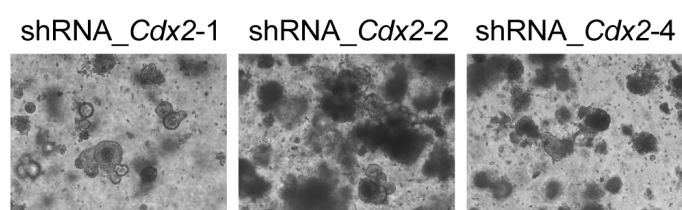

**D**

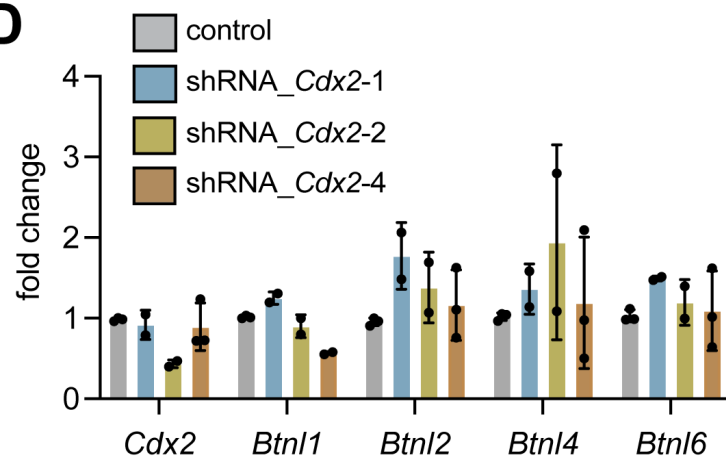

**E**

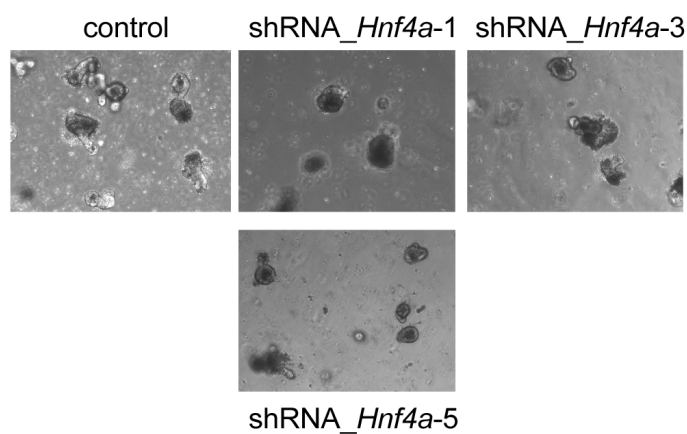

**F**

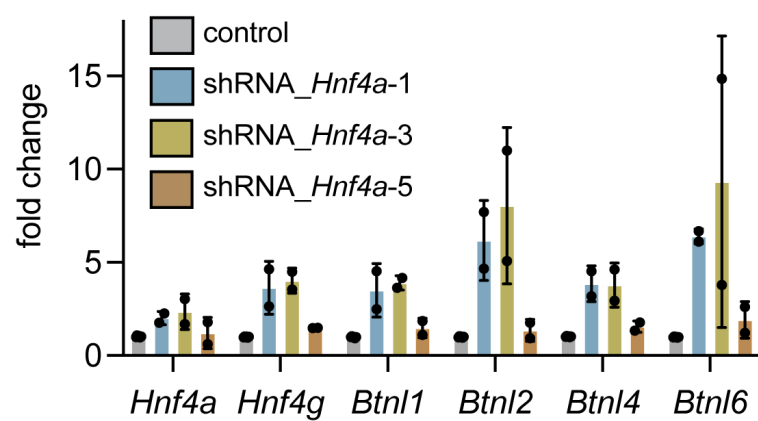

**G**

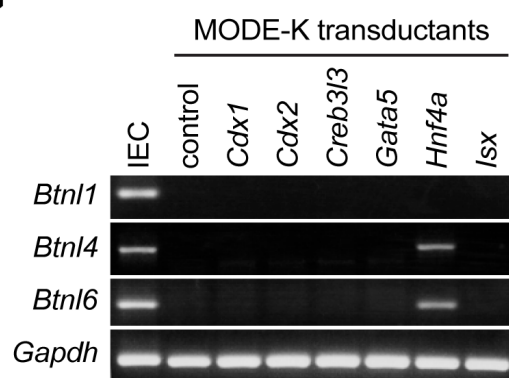

**H**

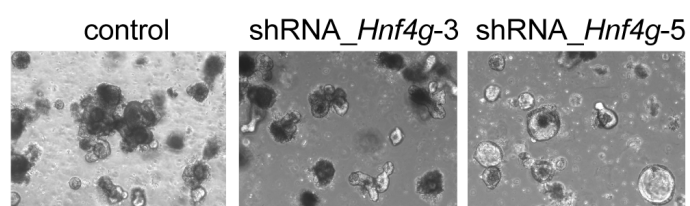

Supplement: Supplemental Figure 3 [file EMS177377-supplement-Supplemental_Figure_3.pdf]

# Supplemental Figure 4

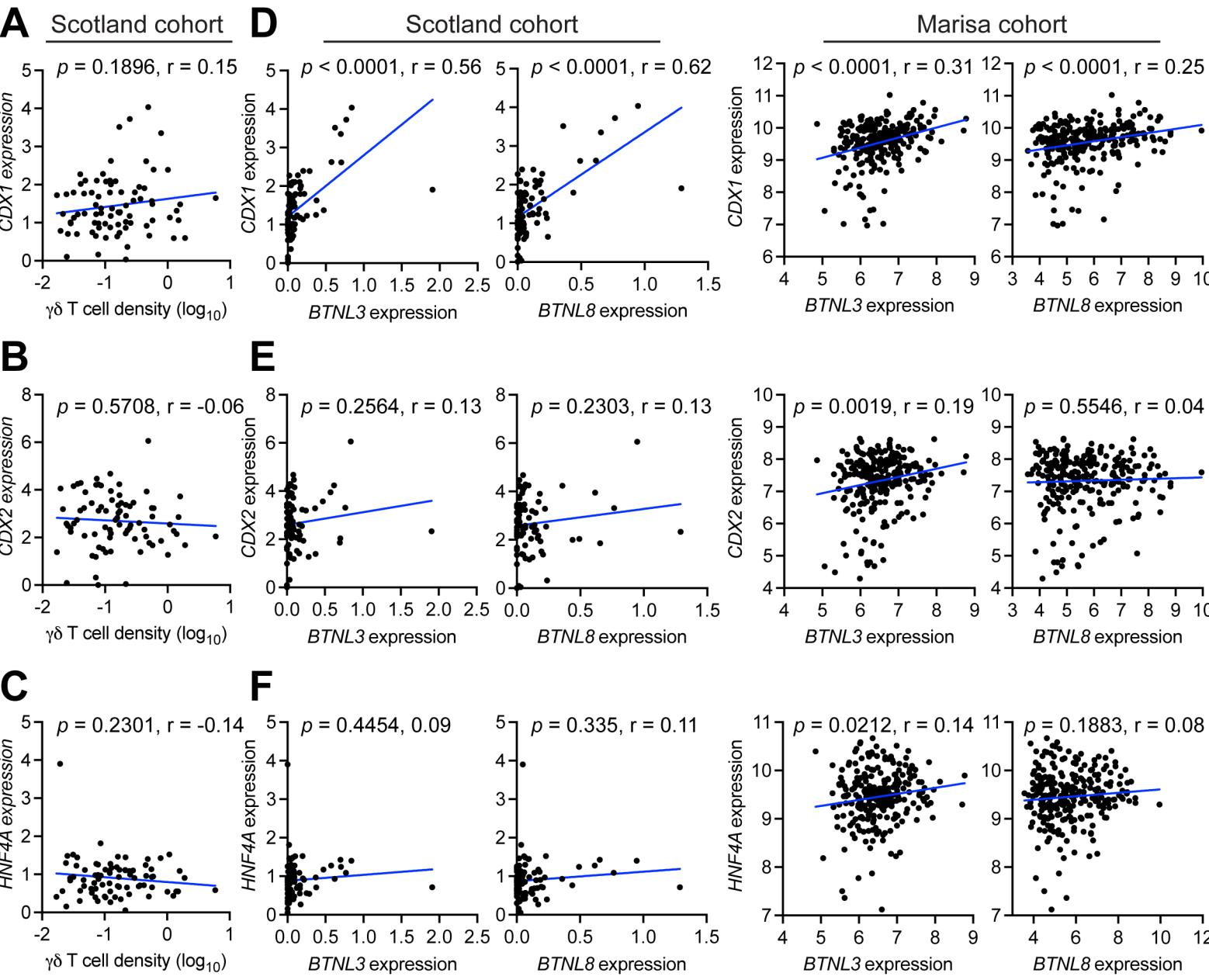

Supplement: Supplemental Figure 4 [file EMS177377-supplement-Supplemental_Figure_4.pdf]

# Supplemental Figure 7

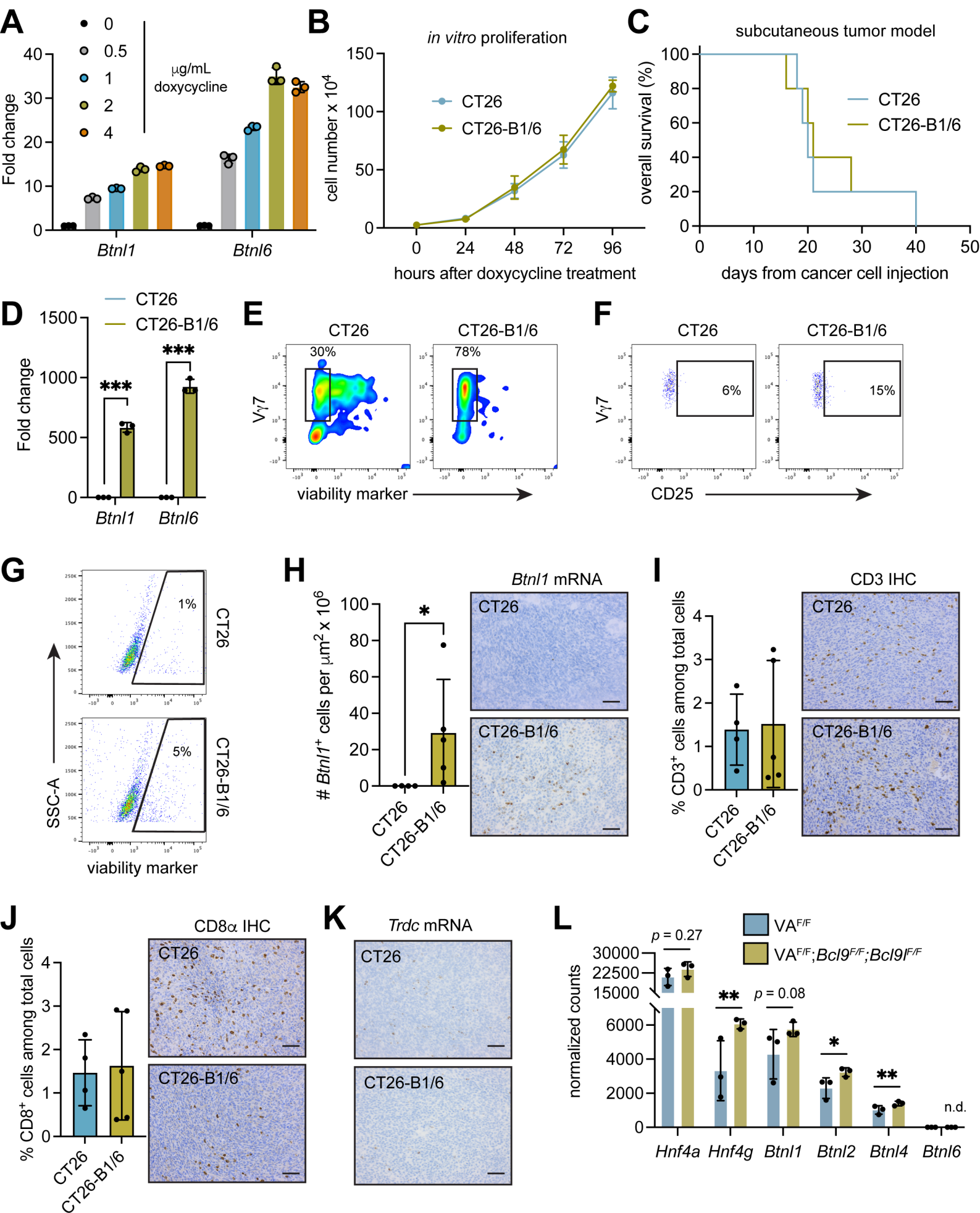

Supplement: Supplemental Figure 7 [file EMS177377-supplement-Supplemental_Figure_7.pdf]
