# Supplementary material for: β-catenin drives butyrophilin-like molecule loss and γδ T-cell exclusion in colon cancer
Source: Cancer Immunol Res. Author manuscript; Available in PMC 2023 Aug 4. (PMC10398359; doi:10.1158/2326-6066.CIR-22-0644)

Supplemental Figure 5

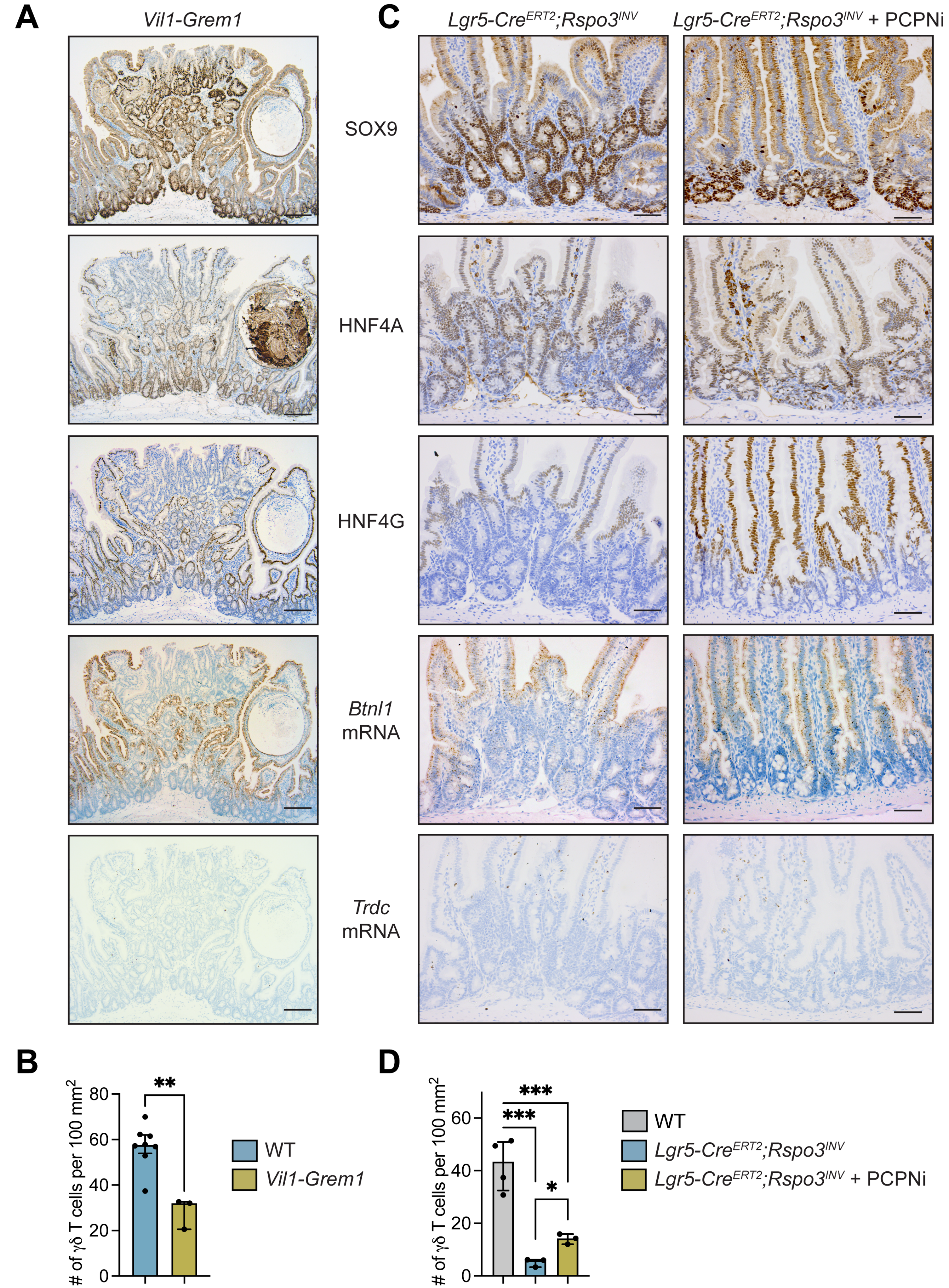

Supplement: Supplemental Figure 5 [file EMS177377-supplement-Supplemental_Figure_5.pdf]
