# Supplementary material for: β-catenin drives butyrophilin-like molecule loss and γδ T-cell exclusion in colon cancer
Source: Cancer Immunol Res. Author manuscript; Available in PMC 2023 Aug 4. (PMC10398359; doi:10.1158/2326-6066.CIR-22-0644)

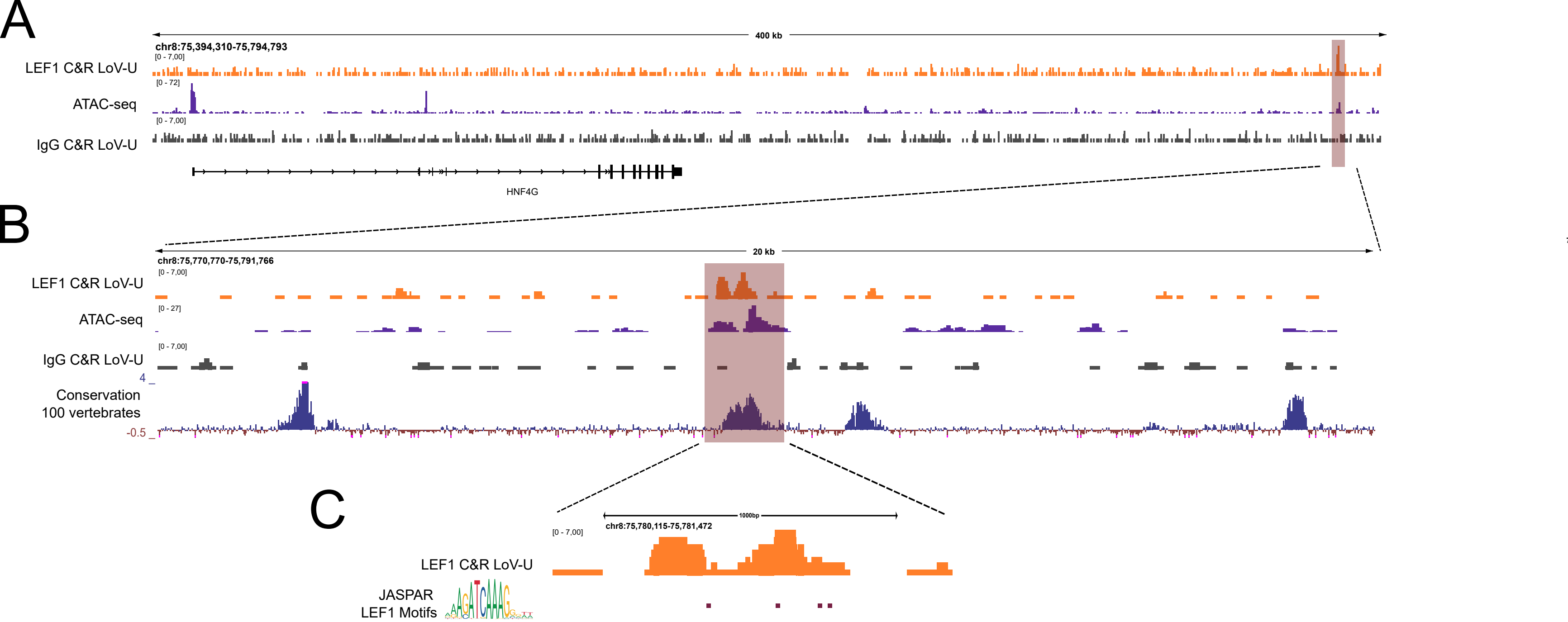

D

GREAT version 4.0.4 current (08/19/2019 to now)

All genomic region-gene association tables (1 regions, 2 genes)

Genomic region -> gene association table

Region	Gene (distance to TSS)
WRE	ZFHX4 (-900,450), HNF4G (+240,900)

Gene -> genomic region association table

Gene	Region (distance to TSS)
HNF4G	WRE (+240,900)
ZFHX4	WRE (-900,450)

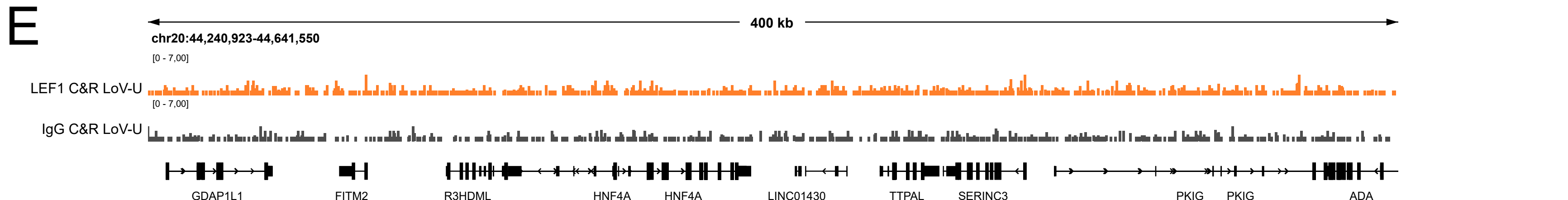

Supplement: Supplemental Figure 6 [file EMS177377-supplement-Supplemental_Figure_6.pdf]
