# Supplementary material for: β-catenin drives butyrophilin-like molecule loss and γδ T-cell exclusion in colon cancer
Source: Cancer Immunol Res. Author manuscript; Available in PMC 2023 Aug 4. (PMC10398359; doi:10.1158/2326-6066.CIR-22-0644)
Supplement: Supplementary legend [file EMS177377-supplement-Supplementary_legend.pdf]

## SUPPLEMENTIONAL INFORMATION

**Supplemental Figure S1. WNT expression is independent of *BTN3A1* and *BTN2A1.***

(A) Correlation between *CTNNB1* or *SOX9* expression and *BTN3A1* or *BTN2A1* expression. Units on axes are normalized counts x $10^3$. Each dot represents one tumor (n = 82 Scotland cohort). *P* value and r value determined by Pearson's correlation.

**Supplemental Figure S2. Deletion of *Apc* in organoids increases WNT target genes.**

(A) Fold change in expression levels of indicated genes in WT, $VA^{F/F}$, VK and $VA^{F/F}K$ organoids. Gene expression was measured at indicated days post tamoxifen treatment. Each dot represents one organoid from one mouse (n = 3). Data presented as mean ± SD. *$p < 0.05$, **$p < 0.01$ and ***$p < 0.001$ as determined by one-way ANOVA followed by Dunnett's posthoc test.

**Supplemental Figure S3. Knockdown of *Cdx1, Cdx2* and *Hnf4a* fails to influence organoid morphology or expression of *Btnl* genes.**

(A) Representative images of organoids from WT mice transduced with shRNA constructs against *Cdx1*. (B) Fold change in expression levels of indicated genes in WT organoids transduced with shRNA constructs targeting *Cdx1* transcripts. Each dot represents one organoid from one mouse (n = 3). Data presented as mean ± SD. ***$p < 0.001$ as determined by one-way ANOVA followed by Tukey's posthoc test. (C) Representative images of organoids from WT mice transduced with shRNA constructs against *Cdx2*. (D) Fold change in expression levels of indicated genes in WT organoids transduced with shRNA constructs targeting *Cdx2* transcripts. Each dot represents one organoid from one mouse (n = 2-3). Data presented as mean ± SD. (E) Representative images of organoids from WT mice transduced with shRNA constructs against *Hnf4a*. (F) Fold change in expression levels of indicated genes in WT organoids transduced with shRNA constructs targeting *Hnf4a* transcripts. Each dot represents one organoid from one mouse (n = 2). Data presented as mean ± SD. (G) Representative RT-PCR product bands for MODE-K cells transduced with transcription factor over-expression constructs. Intestinal epithelial cells (IEC) served as positive control, while empty vector (control) served as negative control. (H) Representative images of organoids from WT mice transduced with shRNA constructs against *Hnf4g*.

**Supplemental Figure S4. Correlation between gut-specific transcription factors, $\gamma\delta$ T cell density and *BTNL* genes in human tumors.**

(A) Correlation between *CDX1* expression as determined by TempO-Seq and $\gamma\delta$ T cell density determined by IHC in the Scotland cohort. Units on axes are normalized counts x $10^3$. Each dot

represents one tumor (n = 77). *P* value and r value determined by Pearson's correlation. (B) Correlation between *CDX2* expression as determined by TempO-Seq and γδ T cell density determined by IHC in the Scotland cohort. Units on axes are normalized counts x $10^3$. Each dot represents one tumor (n = 77). *P* value and r value determined by Pearson's correlation. (C) Correlation between *HNF4A* expression as determined by TempO-Seq and γδ T cell density determined by IHC in the Scotland cohort. Units on axes are normalized counts x $10^3$. Each dot represents one tumor (n = 77). *P* value and r value determined by Pearson's correlation. (D) Correlation between *BTNL3* or *BTNL8* expression and *CDX1* expression. Units on axes are normalized counts x $10^3$. Each dot represents one tumor (n = 82 Scotland cohort, 258 Marisa cohort). *P* value and r value determined by Pearson's correlation. (E) Correlation between *BTNL3* or *BTNL8* expression and *CDX2* expression. Units on axes are normalized counts x $10^3$. Each dot represents one tumor (n = 82 Scotland cohort, 258 Marisa cohort). *P* value and r value determined by Pearson's correlation. (F) Correlation between *BTNL3* or *BTNL8* expression and *HNF4A* expression. Units on axes are normalized counts x $10^3$. Each dot represents one tumor (n = 82 Scotland cohort, 258 Marisa cohort). *P* value and r value determined by Pearson's correlation.

**Supplemental Figure S5. Disruption of WNT gradient in normal intestinal villi reduces γδ T cells.**

(A) Representative images of SOX9, HNF4A, HNF4G, *Btnl1* and *Trdc* expression in small intestine from 3 *Vil1-Grem1* mice. Scale bar = 500 μm. (B) Graphic representation of γδ T cell numbers in intestinal tissue of WT and *Vil1-Grem1* mice. Each dot represents one mouse (n = 8 WT, 3 *Vil1-Grem1*). Data presented as mean ± SD per 100 $mm^2$. ***p* < 0.01 as determined by unpaired t test. (C) Representative images of SOX9, HNF4A, HNF4G, *Btnl1* and *Trdc* expression in small intestine from *Lgr5-Cre*$^{ERT2}$*;Rspo3*$^{INV}$ mice treated with vehicle control or LGK-974 (PCPNi). Scale bar = 500 μm. (D) Graphic representation of γδ T cell numbers in intestinal tissue of WT, *Lgr5-Cre*$^{ERT2}$*;Rspo3*$^{INV}$ mice and PCPNi-treated *Lgr5-Cre*$^{ERT2}$*;Rspo3*$^{INV}$ mice. Each dot represents one mouse (n = 4 WT, 3 *Lgr5-Cre*$^{ERT2}$*;Rspo3*$^{INV}$, 3 *Lgr5-Cre*$^{ERT2}$*;Rspo3*$^{INV}$ + PCPNi). Data presented as mean ± SD per 100 $mm^2$. **p* < 0.05, ****p* < 0.001 as determined by one-way ANOVA followed by Tukey's posthoc test.

**Supplemental Figure S6. Identification of a WNT responsive element (WRE) in the *HNF4G* genomic locus.**

(A) HEK293T cells were stimulated with 10 μM CHIR-99021 and analyzed by CUT&RUN Low Volume Urea (C&R LoV-U) for LEF1 binding sites and ATAC-sequencing (ATAC-seq) at the

*HNF4G* locus. This locus is annotated to *HNF4G* according to Genomic Regions Enrichment of Annotations Tool (GREAT). (B) Close view of the LEF1 and ATAC peak region with sequence conservation data from the UCSC genome browser. (C) Depiction of four LEF1 binding motifs (predicted by JASPAR LEF1 core MA0768.1) found within the C&R LEF1 peak region (black squares under orange peaks). (D) GREAT prediction analysis of WRE within genomic regions of chromosome 8 near the *HNF4G* locus. TSS = transcription start site. (E) HEK293T cells were stimulated with 10 μM CHIR-99021 and analyzed by CUT&RUN Low Volume Urea (C&R LoV-U) for LEF1 binding sites and ATAC-sequencing (ATAC-seq) at the *HNF4A* locus. This locus is annotated to *HNF4A* according to GREAT.

**Supplemental Figure S7. Impact of *Btnl1* and *Btnl6* expression on Vγ7$^+$ cells and the tumor microenvironment.**

(A) *Btnl1* and *Btnl6* gene expression in CT26 cells transduced with doxycycline-inducible vectors as determined by qRT-PCR. Cells were treated with doxycycline at the concentration shown for 24 hours. Each dot represents a biological replicate (n = 3). (B) Cell proliferation of doxycycline-treated CT26 and CT26-B1/6 cells over the course of 96 hours. Each dot represents a biological replicate (n = 3). (C) Kaplan-Meier survival analysis of doxycycline-treated mice bearing subcutaneous CT26 or CT26-B1/6 tumors (n = 5 CT26, 5 CT26-B1/6). (D) *Btnl1* and *Btnl6* gene expression in tumors from doxycycline-treated CT26 and CT26-B1/6 tumor-bearing mice as determined by qRT-PCR. Each dot represents one tumor (n = 3/group). ***$p < 0.001$ as determined by unpaired t test. (E) Representative dot plots of Vγ7$^+$ cell viability from co-cultures with doxycycline-treated CT26 or CT26-B1/6 cells *in vitro*. (F) Representative dot plots of CD25 staining on Vγ7$^+$ cells from co-cultures with doxycycline-treated CT26 or CT26-B1/6 cells *in vitro*. (G) Representative dot plots of CT26 or CT26-B1/6 cell viability from co-cultures with Vγ7$^+$ cells *in vitro*. (H) Quantification and representative staining of *Btnl1* mRNA expression in tumors from doxycycline-treated mice bearing CT26 and CT26-B1/6 cells. Each dot represents one mouse (n = 4 CT26, 5 CT26-B1/6). Data presented as mean ± SD. *$p < 0.05$ as determined by unpaired t test. Scale bar = 500 μm. (I) Quantification and representative staining of CD3 expression in tumors from doxycycline-treated mice bearing CT26 and CT26-B1/6 cells. Each dot represents one mouse (n = 4 CT26, 5 CT26-B1/6). Data presented as mean ± SD. Scale bar = 500 μm. (J) Quantification and representative staining of CD8α expression in tumors from doxycycline-treated mice bearing CT26 and CT26-B1/6 cells. Each dot represents one mouse (n = 4 CT26, 5 CT26-B1/6). Data presented as mean ± SD. Scale bar = 500 μm. (K) Representative staining of *Trdc*

mRNA expression in tumors from doxycycline-treated mice bearing CT26 and CT26-B1/6 cells. Scale bar = 500 μm. (L) Gene expression of indicated molecules in $VA^{F/F}$ and $VA^{F/F}$;*Bcl9*$^{F/F}$;*Bcl9l*$^{F/F}$ intestinal tissue generated from RNAseq data. Each dot represents one mouse (n = 3). Data presented as mean ± SD. **p* < 0.05, ***p* < 0.01 as determined by unpaired t test. n.d. = not detected.
