# Supplementary material for: β-catenin drives butyrophilin-like molecule loss and γδ T-cell exclusion in colon cancer
Source: Cancer Immunol Res. Author manuscript; Available in PMC 2023 Aug 4. (PMC10398359; doi:10.1158/2326-6066.CIR-22-0644)
Supplement: Supplementary Table S1 [file EMS177377-supplement-Supplementary_Table_S1.pdf]

Supplementary Table S1. Clinicopathological characteristics of colon cancer patient cohorts.

	Scotland	*Norway*	*Thailand*
**Sex**			
Female	73 (51%)	41 (49%)	60 (44%)
Male	71 (49%)	43 (51%)	76 (56%)
**Age**			
>=65	90 (62%)	68 (81%)	69 (51%)
<65	54 (38%)	16 (19%)	67 (49%)
**Tumor site**			
Right	67 (47%)	48 (57%)	n.a.
Left	46 (32%)	36 (43%)	
Rectum	31 (22%)	0 (0%)	
**TMN Stage**			
I	16 (11%)	0 (0%)	0 (0%)
II	60 (42%)	51 (61%)	24 (18%)
III	63 (44%)	33 (39%)	61 (45%)
IV	5 (3%)	0 (0%)	37 (27%)
**Differentiation**			
Moderate/Well	131 (91%)	65 (77%)	133 (98%)
Poor	13 (9%)	16 (19%)	0 (0%)
Missing	0 (0%)	3 (3.6%)	3 (2.2%)
**MMR Status**			
Proficient	116 (81%)	n.a.	n.a.
Deficient	25 (17%)		
Missing	3 (2.1%)		
